# Histone H3-wild type diffuse midline gliomas with H3K27me3 loss are a distinct entity with exclusive *EGFR* or *ACVR1* mutation and differential methylation of homeobox genes

**DOI:** 10.1038/s41598-023-30395-4

**Published:** 2023-03-07

**Authors:** Pamela Ajuyah, Chelsea Mayoh, Loretta M. S. Lau, Paulette Barahona, Marie Wong, Hazel Chambers, Fatima Valdes-Mora, Akanksha Senapati, Andrew J. Gifford, Colleen D’Arcy, Jordan R. Hansford, Neevika Manoharan, Wayne Nicholls, Molly M. Williams, Paul J. Wood, Mark J. Cowley, Vanessa Tyrrell, Michelle Haber, Paul G. Ekert, David S. Ziegler, Dong-Anh Khuong-Quang

**Affiliations:** 1 Lowy Cancer Research Centre, Children’s Cancer Institute, UNSW Sydney, Kensington, NSW Australia; 2 School of Clinical Medicine, UNSW Medicine & Health, UNSW Sydney, Kensington, NSW Australia; 3 University of New South Wales Centre for Childhood Cancer Research, UNSW, Kensington, NSW Australia; 4 Kids Cancer Centre, Sydney Children’s Hospital, High Street, Randwick, NSW 2031 Australia; 5 Department of Anatomical Pathology, Royal Children’s Hospital, University of Melbourne, Melbourne, VIC Australia; 6 Anatomical Pathology, NSW Health Pathology, Prince of Wales Hospital, Randwick, NSW Australia; 7 Children’s Cancer Centre, Royal Children’s Hospital, 50 Flemington Road, Parkville, VIC 3052 Australia; 8 Murdoch Children’s Research Institute, Royal Children’s Hospital, Parkville, VIC Australia; 9 Department of Paediatrics, University of Melbourne, Parkville, VIC Australia; 10 Michael Rice Cancer Centre, Women’s and Children’s Hospital, Adelaide, SA Australia; 11 South Australia Health and Medical Research Institute, Adelaide, SA Australia; 12 South Australia Immunogenomics Cancer Institute, Adelaide, SA Australia; 13 University of Adelaide, Adelaide, SA Australia; 14 Oncology Service, Children’s Health Queensland Hospital & Health Service, Brisbane, QLD Australia; 15 Department of Paediatrics, School of Clinical Sciences at Monash Health, Monash University, Clayton, VIC Australia; 16 Cancer Immunology Program, Peter MacCallum Cancer Centre, Parkville, VIC Australia; 17 The Sir Peter MacCallum Department of Oncology, University of Melbourne, Parkville, VIC Australia

**Keywords:** Cancer, Genetics

## Abstract

Diffuse midline gliomas (DMG) harbouring H3K27M mutation are paediatric tumours with a dismal outcome. Recently, a new subtype of midline gliomas has been described with similar features to DMG, including loss of H3K27 trimethylation, but lacking the canonical H3K27M mutation (H3-WT). Here, we report a cohort of five H3-WT tumours profiled by whole-genome sequencing, RNA sequencing and DNA methylation profiling and combine their analysis with previously published cases. We show that these tumours have recurrent and mutually exclusive mutations in either *ACVR1* or *EGFR* and are characterised by high expression of *EZHIP* associated to its promoter hypomethylation. Affected patients share a similar poor prognosis as patients with H3K27M DMG. Global molecular analysis of H3-WT and H3K27M DMG reveal distinct transcriptome and methylome profiles including differential methylation of homeobox genes involved in development and cellular differentiation. Patients have distinct clinical features, with a trend demonstrating *ACVR1* mutations occurring in H3-WT tumours at an older age. This in-depth exploration of H3-WT tumours further characterises this novel DMG, H3K27-altered sub-group, characterised by a specific immunohistochemistry profile with H3K27me3 loss, wild-type H3K27M and positive EZHIP. It also gives new insights into the possible mechanism and pathway regulation in these tumours, potentially opening new therapeutic avenues for these tumours which have no known effective treatment.

This study has been retrospectively registered on clinicaltrial.gov on 8 November 2017 under the registration number NCT03336931 (https://clinicaltrials.gov/ct2/show/NCT03336931).

## Introduction

High grade gliomas (HGG) remain a leading cause of death in childhood, with a five-year overall survival of less than 20% despite aggressive therapeutic approaches. The advent of next generation sequencing has defined distinct genomic profiles that cluster with age at presentation and anatomical location^1,2^. About half of these tumours occur in midline locations^3^, and unique recurrent K27M missense mutations in genes encoding the histone H3 characterise approximately 84% of diffuse intrinsic pontine gliomas and 60% of other midline tumours^2^. Thus, they were defined as a new entity, called *diffuse midline glioma, H3K27M-mutant* (H3K27M DMG) in the 2016 WHO Classification of Tumours of the Central Nervous System (CNS)^4^. Importantly H3K27M mutations confer a shorter survival compared with tumours wild-type for histone H3^2,5^.

The H3K27M mutation exerts its effect in part via loss of histone H3K27 trimethylation (H3K27me3). Recent studies have identified a subset of midline gliomas with wild-type histone H3 (H3-WT), but with concomitant loss of histone H3K27me3^6–9^. Interestingly, in this subgroup, H3-WT tumours express high levels of EZH Inhibitory Protein (*EZHIP*) previously known as *CXorf67*^6,8,9^. EZHIP is a protein primarily expressed in gonads^10^, and reported to be highly expressed in posterior fossa group A ependymoma (PFA-EPD)^11^, where it acts similarly to the H3K27M oncohistone mutant, inhibiting the PRC2 complex^12,13^. The driver of *EZHIP* over-expression in wild-type DMG remains unknown. Previous publications by Pratt et al.^9^, Mondal et al.^7^, Castel et al.^6^ and Sievers et al.^8^ identified more than twenty midline HGG patients with wild-type histone H3 genes and loss of H3K27me3 by immunohistochemistry (IHC). One study found that bi-thalamic H3-WT tumours with loss of H3K27me3 often harboured Epidermal Growth Factor Receptor (*EGFR)* mutations^7^, while another suggested Activin A Receptor Type 1 (*ACVR1)* might be co-mutated with *EZHIP* overexpression^6^. Reflecting on these novel findings, a new entity was introduced in the most recent WHO 2021 classification, *diffuse midline glioma, H3 K27-altered*^14^, which includes both the H3-WT subset and those with H3K27M mutation.

Here, we build on these initial reports by performing a comprehensive genomic analysis on 5 patients with DMGs lacking the H3K27M mutation but with a loss of H3K27me3, and up-regulation of *EZHIP*. We show that these tumours can be divided into distinct subgroups based on their genomic profile and define their clinical outcome. Strikingly, we found a plausible mechanism by which *EZHIP* is over-expressed in these samples, potentially through promoter DNA hypomethylation. In addition, further exploration of the DNA methylome in these patients revealed significant changes that correlate with dysregulation of developmental genes including several homeobox gene family members.

## Materials and methods

### Patients and samples

Patients were enrolled on the PRecISion Medicine for Children with Cancer clinical trial (NCT03336931), as part of the Australian Zero Childhood Cancer (ZERO) Precision Medicine Program. ZERO is an Australian national paediatric precision medicine program currently focused on real time recruitment and analysis of patients with high-risk paediatric cancers (< 30% chance of survival). Informed consent was provided by the parents/legal guardian for participants under the age of 18 years and by participants over the age of 18 years^15^. Eighty-nine patients diagnosed with brain tumours were enrolled on the ZERO clinical trial from September 2017 until May 2020. Amongst these patients, 28 were diagnosed with a H3K27M DMG and 39 with other high-grade glioma lacking the H3K27M mutation (HGG), including WHO grade III anaplastic astrocytoma and grade IV glioblastomas (GBM) irrespective of their anatomical location or their molecular profile besides the absence of H3K27M mutation^15^. Out of the five cases presented in this study, two cases, zcc120 and zcc183 were previously reported in part^15^.

The molecular profiling platform consisted of germline and tumour whole genome sequencing (WGS) associated with matched germline DNA WGS, tumour only RNA-sequencing and tumour DNA Infinium MethylationEPIC array (Illumina). DNA and RNA were extracted from fresh, fresh frozen or cryopreserved tumour tissue and matched germline samples (from either fresh, cryopreserved or fresh frozen peripheral blood or skin) at the Children’s Cancer Institute (Australia), as described previously^15^. WGS was conducted at the Kinghorn Centre for Clinical Genomics at the Garvan Institute of Medical Research (Australia), DNA methylation array performed by the Australian Genome Research Facility and transcriptome sequencing performed at Murdoch Children’s Research Institute (Australia).

Additional cohorts were used in this study from Mondal et al.^7^ GSE140124 (N = 9 H3-WT cases) and Castel et al.^6^ E-MTAB-8888 (N = 14 H3-WT and N = 25 H3.3-K27M and H3.1-K27M mutant cases).

### Sequencing and molecular analyses

Full details of the methodology have been described previously^15^. In brief, DNA libraries were prepared using either TruSeq Nano HT Sample Prep Kit (Illumina) or KAPA PCR-Free v2.1 (Roche) and sequenced on the Illumina HiSeq X Ten platform. Germline samples were sequenced to an average depth of 30X and tumour samples 90X. RNA libraries used the TruSeq Stranded mRNA Preparation Kit and sequenced on either the HiSeq 4000 or NextSeq 500 platform to a targeted paired-end read depth of 80 M reads.

WGS germline variants were subtracted from the tumour sequencing data to identify somatic only variants. Somatic variants were individually analysed with prioritisation of variants in cancer related genes and were manually mined for any mutations containing K27M, K27I, G34R, G34V or G34W changes in the following histone genes: *H3F3A, H3F3B, H3F3C, HIST1H1A, HIST1H1B, HIST1H2AA, HIST1H2BA, HIST1H3A, HIST1H4A, HIST1H3C, HIST2H3C, HIST3H2BB* and *HIST3H3*. Genes involved in the PRC2 complex and common epigenetic regulators were also mined for molecular aberrations in *DNMT1, DNMT3A, DNMT3B, DNMT3L, TET1, TET2, TET3, EED, EZH2, SUZ12, SET, RBAP, KDM6A, BCOR, BCORL1, CREBBP, LZTR1* and *ASZL1*.

### Immunohistochemistry

IHC for trimethylated H3K27me3 (1:100, Cat # 07–449, Millipore), mutant H3K27M (1:1000, Cat # ABE419, Millipore), EZHIP (1:200, Cat # HPA004003, Sigman-Aldrich) was performed on formalin-fixed, paraffin embedded (FFPE) tissue as previously described^16,17^, on the Ventana BenchMark ULTRA IHC/ISH system for all antibodies. Detailed protocols are available upon request. The results were assessed by anatomical pathologists (CD, AJG). Positive staining for H3K27M was defined as nuclear staining within tumours cells. The presence or absence of nuclear H3K27me3 immunoreactivity was also assessed. Positive EZHIP expression was defined by strong and diffuse nuclear staining within tumour cells. All stains were run with relevant positive and negative controls.

### Methylation analysis

DNA Infinium Methylation EPIC 850K Array Data was analysed in R (v4.0.5) (https://www.R-project.org/) using Bioconductor package limma (v3.46.0)^18^ and minfi (v1.36.0)^19^ for quality control checks, removal of poor performing probes, batch correction, normalisation, calculation of the beta and M values for each probe and data was annotated against the hg19 genome build. Beta values closer to 1 correspond with the probe being methylated and beta values closer to 0 unmethylated. Differential methylation analysis was performed using limma and significantly differentially methylated probes were considered if they had an adjusted p-value < 0.05 and were located within a CpG Island, resulting in 277 significant genes. Gene Ontology (GO) enrichment analysis^20^ was performed on this list of significantly differentially methylated genes. All methylation samples were processed through the Molecular Neuropathology CNS classifier (v11b4 and v12.5)^21^ for tumour classification against more than 80 tumour classes and subclasses.

### Statistical analysis

t-SNE (t-Distributed Stochastic Neighbour Embedding) analysis was performed using Rtsne (v0.15) for both transcriptome and methylome unsupervised clustering analysis (https://github.com/jkrijthe/Rtsne). Combined methylation clustering analysis on the ZERO, GSE140124 and E-MTAB-8888 datasets was performed on only the probes that overlap between the Illumina 450K and EPIC arrays. Overall survival curves were analysed using the Kaplan–Meier method and the log-rank test was used to make univariate assessments of Kaplan–Meier plots. p value ≤ 0.05 was considered significant.

### Ethics approval and consent to participate

This study was conducted according to the guidelines of the Declaration of Helsinki and approved by the Hunter New England Human Research Ethics Committee of the Hunter New England Local Health District (reference no. 17/02/015/4.06) and the New South Wales Human Research Ethics Committee (reference no. HREC/17/HNE/29). Informed consent for each participant was provided by legal guardian for participants under the age of 18 years and by the participants who were over the age of 18.

## Results

### EZHIP is over-expressed in a subset of high-grade gliomas that cluster with diffuse midline gliomas

From the eighty-nine patients with brain tumours enrolled in the Australian ZERO program, we identified 5 patients presenting with the typical clinical, radiological, histological (anaplastic astrocytoma or GBM) and immunohistochemical (loss of H3K27me3) features of a H3K27M DMG, but without histone H3 mutations (Table 1 and Supplementary Fig. 1a–c).

**Table 1:** Clinical and molecular characteristics of H3-WT patients from ZERO.

Patient ID	zcc120	zcc183	zcc316	zcc339	zcc446
Age(years)/gender	15/F	3/M	1/M	8/M	13/M
Histology	Glioblastoma	Anaplastic astrocytoma	Anaplastic astrocytoma	Glioblastoma	Anaplastic astrocytoma
Location	Thalamus, left	Thalamus, bilateral	Thalamus, left	Thalamus, right	Pons, left
IHC	H3K27M negative, loss of H3K27me3 nuclear staining, EZHIP positive	H3K27M negative, loss of H3K27me3 nuclear staining, EZHIP positive	H3K27M negative, loss of H3K27me3 nuclear staining, EZHIP N/A	H3K27M negative, loss of H3K27me3 nuclear staining, EZHIP positive	H3K27M negative, loss of H3K27me3 nuclear staining, EZHIP negative
Tumour purity (%)	94	91	82	90	84
Genetic alterations	NM_001105(ACVR1):c.983G > A (p.Gly328Glu); NM_006218(PIK3CA):c.1633G > A (p.Glu545Lys)	NM_005228(EGFR):c.2303_2311dupGCGTGGACA (p.Ser768_Asp770dup); NM_005378(MYCN):c.131C > T (p.Pro44Leu)	NM_005228(EGFR):c.2303_2311dupGCGTGGACA (p.Ser768_Asp770dup)	NM_001105(ACVR1):c.983G > T (p.Gly328Val); NM_006218(PIK3CA):c.1624G > A (p.Glu542Lys)	NM_000267(NF1):c.574C > T (p.Arg192Ter); NM_001126112(TP53):c.148_157delATTGAACAAT (p.Ile50GlyfsTer70)
Copy number alterations	1p loss, 1q gain, chr2 gain, focal 14q gain/loss	1q/19p co-polysomy	Chr3 gain	1p loss, 1q gain, 15q loss	Focal 1p gain, 1q gain, chr2 gain, 3p loss, focal 4q gain, chr5 gain, focal 9q loss/gain, chr7 gain – includes EGFR (TPM 35.77, FC = 2.71), 8q gain, focal 9p biallelic loss, 10p gain, focal 11p loss, 11q gain, 12p gain and focal amp, 12q focal gain, 13q focal gains and losses, 16p gain, 17p loss
*EZHIP* expression (TPM)	7.57	3.96	3.64	9.51	0.29
DKFZ methylation classifier findings (v11b.4)	No match: methylation class diffuse midline glioma H3 K27M mutant (0.37)	No match: methylation class family glioblastoma, IDH wildtype (0.84)	No match	No match: methylation class family Glioblastoma, IDH wildtype (0.68)	No match: methylation class family glioblastoma, IDH wildtype (0.86)
DKFZ methylation classifier findings (v12.5)	No match: Medulloblastoma, SHH-activated, subclass 2 (novel) (0.43)	Match: diffuse midline glioma, H3 K27-altered, subtype EGFR-altered (0.99)	Match: diffuse midline glioma, H3 K27-altered, subtype EGFR-altered (0.99)	Match: diffuse midline glioma, H3 K27-altered, subtype H3 K27-mutant or EZHIP expressing (0.97)	No match: glioblastoma, IDH-wildtype (0.71)
	No match: Diffuse pediatric-type high-grade glioma, H3-wildtype and IDH-wildtype (0.34)				No match: glioblastoma, IDH-wildtype, subtype posterior fossa (novel) (0.27)
WHO 2021 diagnosis	Diffuse midline glioma, H3 K27-altered	Diffuse midline glioma, H3 K27-altered	Diffuse midline glioma, H3 K27-altered	Diffuse midline glioma, H3 K27-altered	Diffuse paediatric type high grade glioma, H3-wildtype and IDH-wildtype
Radiotherapy	Focal	Focal	Focal (30 Gy)	Focal (59.4 Gy)	Focal (59 Gy)
Chemotherapy	Temozolomide/lomustine	Dasatinib (as part of clinical trial) Temozolomide/lomustine	Carboplatin/etoposide/cyclophosphamide	Temozolomide; temozolomide/bevacizumab	Trametinib; ACT001; abemaciclib
Outcome	Died of disease	Died of disease	Alive with progressive disease	Alive with progressive disease	Died of disease
Follow-up (months)	21	11	12	20	11

The five H3-WT midline gliomas with their corresponding zcc ID, age at diagnosis, gender (M-male, F-female), histology, location in the brain, IHC results, genetic alterations and copy number alterations as identified by WGS, *EZHIP* TPM expression as identified by RNA-seq, DKFZ methylation classification results, type of radiation and chemotherapy received, disease outcome, and time in month of last known follow-up.

Clinicopathological features for these patients included a median age of 8 years [range 1–15 years] with a sex ratio M:F of 4:1; all tumours were located in midline brain structures (pons or thalamus) and were either unilateral or bilateral. All patients were treated with focal radiation therapy and a variety of chemotherapy regimens including temozolomide with lomustine or bevacizumab, dasatinib and trametinib (Table 1).

**Figure 1:**
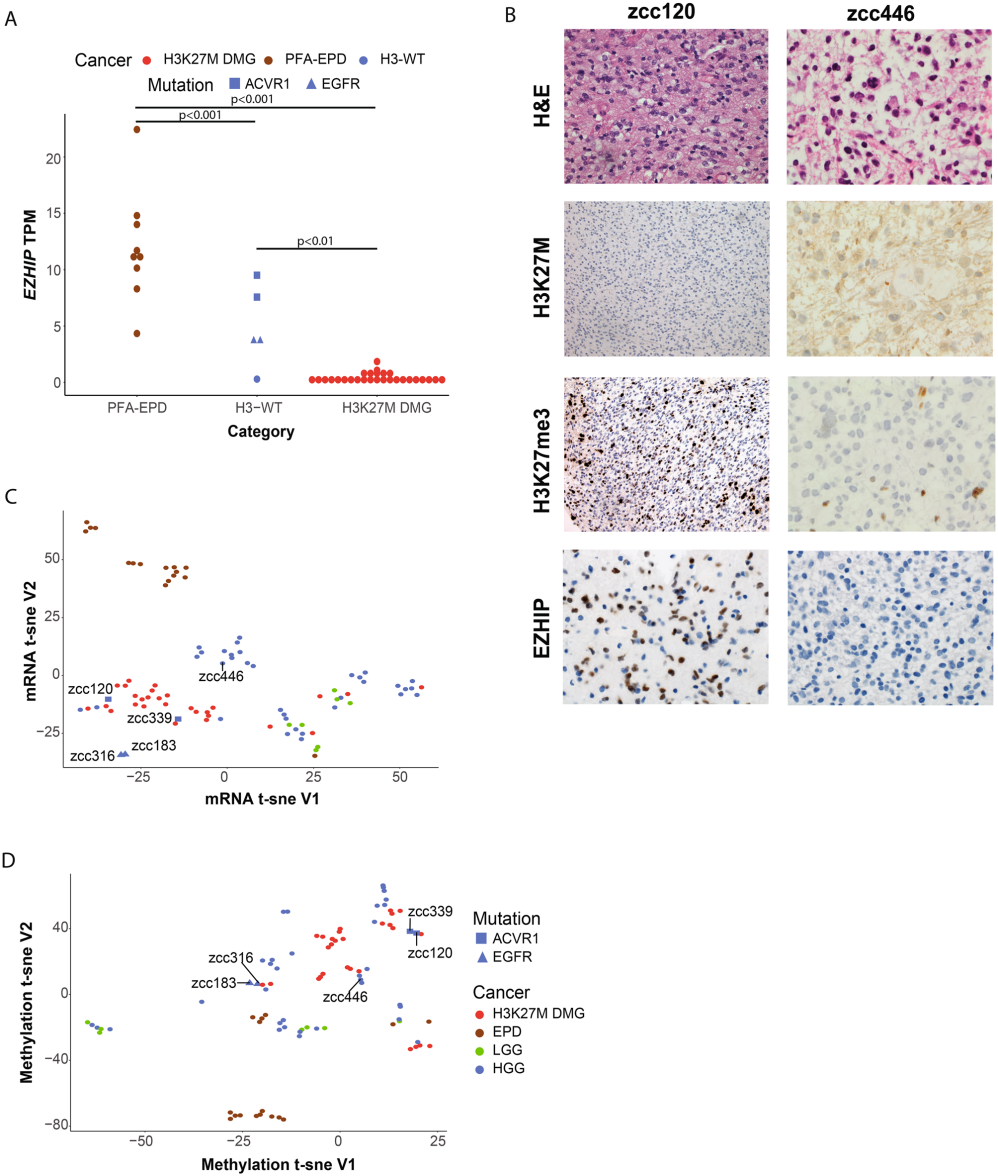
H3-WT patients over-express *EZHIP* and cluster more closely to diffuse midline gliomas. (**A**) Relative expression of *EZHIP* (transcript per million (TPM)) in PFA-EPD, H3-WT midline gliomas and H3K27M DMG. (**B**) H&E and immunohistochemistry panel of zcc120 and zcc446 including H&E (400x, 600x, respectively), H3K27M (200x, 600x, respectively), H3K27me3 (200x, 600x, respectively) and EZHIP (400x, 600x, respectively). For zcc120 (left hand panels), immunohistochemical staining demonstrates negative nuclear staining for H3K27M mutant, loss of nuclear staining for H3K27me3 in tumour cells with preserved staining in inflammatory cells, and nuclear expression EZHIP in tumor cells. For zcc446 (right hand panels), immunohistochemical staining demonstrates negative nuclear staining for H3K27M mutant, loss of nuclear staining for H3K27me3 and absent EZHIP nuclear staining. (**C**) t-SNE unsupervised clustering of transcriptome profile and (**D**) methylation profile of all glioma tumours in the ZERO cohort. For a-c patients are highlighted as H3K27M DMG (N = 28; red), PFA-EPD in a and all EPD in b-c (N = 16; brown), LGG (N = 7; green), and HGG (N = 38; blue).

WGS was performed on all patients and no cryptic or sub-clonal histone alterations, mutations in the PRC2 complex, or genes associated with epigenetic regulation were identified. The tumour mutation burden was universally low (range [0.38–2.16 mutations/Mb]; median 0.94). In addition, 4 out of the 5 cases harboured mutations commonly found in DMG^22–26^, including either *ACVR1* p.G328V or an 11 base pair insertion in *EGFR* exon 20 (Table 1). Consistent with recent reports^6,8^, these 4 cases also expressed high *EZHIP* RNA expression, at levels comparable to PFA-EPD from the ZERO cohort, and higher than in H3K27M DMGs (Fig. 1A). Furthermore, the H3-WT tumours with *ACVR1* mutations had higher expression levels of *EZHIP* than H3-WT tumours with *EGFR* mutations (Fig. 1A). Interestingly, the genome of these 4 cases were overall stable, with very few copy number alterations and structural variants (Supplementary Fig. 2a–d). However, one H3-WT tumour without *EZHIP* overexpression (zcc446) was aneuploid with a high density of single nucleotide and structural variants and lacking either *ACVR1* or *EGFR* mutations (Supplementary Fig. 2e, Table 1).

We confirmed the overexpression of *EZHIP* by immunohistochemistry in the four tumours for which FFPE tissue was available. In keeping with the RNA-seq data, zcc446 staining was negative, while the other tumours showed strong nuclear positivity (Fig. 1B, Supplementary Fig. 1c).

Unsupervised clustering of the transcriptome showed a clear separation of the different glioma subtypes where the *EZHIP*-high H3-WT tumours clustered more closely with DMGs than with *EZHIP*-high EPD samples and the tumour without *EZHIP* up-regulation (zcc446) clustered separately from the DMG cohort and closer to HGGs (Fig. 1C). While the two *ACVR1* mutant cases clustered within the DMG group, the *EGFR* mutants clustered separately from them (Fig. 1C).

In previous analyses, the methylation profiles of H3-WT tumours with H3K27me3 loss clustered with HGG^7^ or DMG^6^ subgroups. Unsupervised clustering of the global DNA methylomes of the 89 CNS tumours from the ZERO cohort (N = 6 low grade gliomas (LGG), N = 16 EPD, N = 28 DMG and N = 39 HGG) confirmed that these tumours cluster separately from EPD and together with HGG and DMG (Fig. 1D). DNA methylation-based clustering also separated H3-WT *EGFR* mutants and H3-WT *ACVR1* mutants as two distinct entities (Fig. 1D). The H3-WT case without *EZHIP* over-expression was distinct from the other 4 H3-WT (Fig. 1C,D). Our data shows that the unique transcriptome and methylome profiles of these glioma subtypes can be used to molecularly classify these tumours.

To further characterise the 5 H3-WT, we next ran our samples through a published CNS methylation-based classifier (v11b4)^21^. None of the 5 cases had a strong match (score > 0.9) with the entities reported in this classifier (Table 1). However, using an updated version of the classifier (v12.5), 3 of the 5 H3-WT tumours were a strong match (> 0.9) for Diffuse midline glioma, H3 K27-altered group. The two cases harbouring *EGFR* mutations were classified within the EGFR-altered subtype (zcc183 and zcc316) and one of the cases with *ACVR1* mutation was classified within the H3 K27-mutant or EZHIP-expressing subtype (zcc339). Of note, the other *ACVR1*-mutated case did not match with any group (zcc120), as well as the H3-WT tumour with low *EZHIP* expression (zcc446), emphasising the potential for further classification refinement.

**Figure 2:**
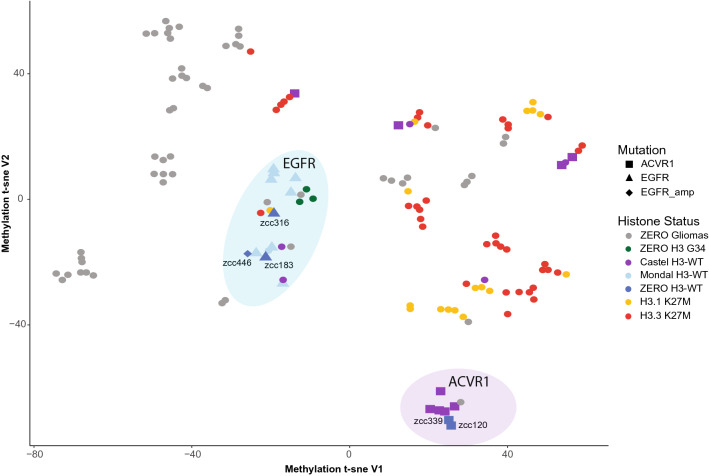
Methylation profiles show segregation between mutant ACVR1 and mutant EGFR H3-WT. t-SNE unsupervised clustering of the methylation profiles of the ZERO gliomas (N = 53; grey), ZERO H3 G34 mutants (N = 3; green), Castel (N = 14), Mondal (N = 9) and ZERO H3-WT (N = 5) midline glioma cohorts (purple, light blue and blue, respectively), and H3.1 K27M (N = 17) and H3.3 K27M (N = 37) (yellow and red, respectively) from all three cohorts.

To facilitate a more comprehensive genomic analysis, we expanded our dataset to include previously published H3-WT cases with available methylation data from Castel et al.^6^ (N = 14) which predominantly contain *ACVR1* mutants and Mondal et al.^7^ (N = 9) cases harbouring *EGFR* mutants, referred to separately as the Castel and Mondal cohorts throughout. Unsupervised clustering of the methylome segregated H3-WT samples into two distinct subgroups as previously observed, with *EGFR* mutant cases clustering together and remaining distinct from *ACVR1* mutant cases (Fig. 2). The H3-WT case, zcc446, without over-expression of *EZHIP* had *EGFR* copy gain and clustered within the H3-WT *EGFR* subgroup. Interestingly, HGGs harbouring histone H3 G34R mutations were also part of this subgroup (Fig. 2). Of note, because of the low number of EPD, LGG and HGG without H3 G34 mutant cases (N = 53 total) preventing clear subgrouping on t-SNE plot, as well as the selection bias for particularly aggressive LGG with expected poor patient outcome in our cohort, we elected to label these tumours all together as ZERO Gliomas. Whilst *EGFR* and *ACVR1* mutant cases clustered distinctly, it is important to note that 4 *ACVR1* mutant cases from the Mondal cohort did not cluster within the *ACVR1* sub-group highlighted, potentially suggesting that there are additional drivers within these cases (Fig. 2). In addition, whilst we noticed a minor separation between H3.1 and H3.3 K27M cases, these were not mutually exclusive, and performing feature selection to identify topmost variable methylated probes as performed in Caper et al.^21^ prior to unsupervised clustering would provide a more distinct clustering of H3K27M specific mutations. However, by performing unsupervised global methylation clustering with combining the ZERO, Mondal and Castel cohorts further strengthens the distinct nature and mutual exclusivity of *ACVR1* and *EGFR* mutated H3-WT cases.

### ACVR1 and EGFR mutations are mutually exclusive in H3-WT cases

**Figure 3:**
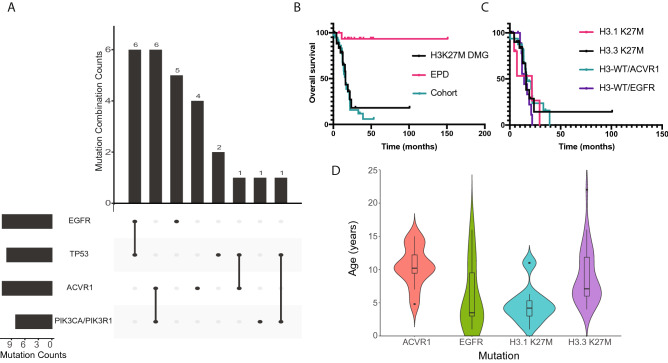
Mutational profiles and clinical outcomes in DMG. (**A**) UpSet plot of the intersection of the four predominant mutations (*EGFR, TP53, ACVR1, PIK3CA/PIK3R1*) observed in H3-WT midline gliomas. (**B**) Kaplan–Meier overall survival analysis comparing H3K27M DMG (N = 27), EPD (N = 16) and the glioma tumour cohort (N = 38). (**C**) Kaplan–Meier overall survival analysis comparing the mutations of interest in this study, respectively H3.3 K27M (N = 21), H3.1 K27M (N = 6), H3-WT *ACVR1* (N = 16) and H3-WT *EGFR* (N = 11). (**D**) Violin plot showing the age distribution of H3-WT *ACVR1* (N = 11), H3-WT *EGFR* (N = 10), H3.1 K27M (N = 18), and H3.3 K27M (N = 36). Horizontal line is the median age and the ends of the box represent the upper and lower quartiles. A black dot indicates the age being greater than two standard deviations from the mean age of the group.

In addition to the *ACVR1* (N = 2) and *EGFR* (N = 2) mutations described above, pathogenic variants in the 5 cases from ZERO were also identified in *PIK3CA* (N = 2), *NF1* (N = 1), *MYC* (N = 1) and *TP53* (N = 1) (Table 1). When combining the ZERO and external cohorts, *ACVR1* and *EGFR* mutations were mutually exclusive. *EGFR* mutations occurred in 11 tumours and mostly co-occurred with *TP53* in 6 of these tumours, whilst *ACVR1* mutations were also present in 11 tumours and co-occurred with *PIK3CA/PIK3R1* mutations in 6 (Fig. 3A). This differential association has similarly been previously reported with the co-occurrence of histone H3.1 K27M, *ACVR1* and/or PI3K mutations, in contrast to co-occurring histone H3.3 K27M and *TP53* mutations^2,25–28^. Furthermore, the *ACVR1* mutations reported in the H3-WT cohort (codons 206, 258, 328 and 356) are seen in H3.1 K27M tumours^27^ with the exception of a R307L mutation which to our knowledge has only been reported in the context of heart defects^29^.

In a combined analysis, patients with H3-WT tumours had an overall survival similar to patients with H3K27M DMGs, which was dismal compared to patients with PFA-EPD (p = 0.57 and p ≤ 0.0001 respectively; Fig. 3B). No differences in survival were noted between H3.3 K27M DMGs, H3.1 K27M DMGs, and H3-WT irrespective of *ACVR1* or *EGFR* mutation (p = 0.46; Fig. 3C), with the caveat of small cohort sizes. Moreover, there was a trend towards patients with H3-WT tumours and *ACVR1* mutations being older than patients with H3-WT and *EGFR* mutant tumours (median = 10 years [range 5–15] and median = 3.5 years [range 1–16], respectively), although no significant difference was observed (p = 0.265; Fig. 3D). They were also older than patients with H3.1 K27M (median = 4 years [range 1–46]) and older than patients with H3.3 K27M (median = 7.5 years [range 4–22]). Interestingly, the median age of patients with *EGFR* mutant H3-WT was comparable to H3.1 K27M DMG patients. As expected, patients with H3.3 K27M DMGs were older than patients with H3.1 K27M DMG^30^ (Fig. 3D).

### EZHIP expression correlates with promoter DNA methylation

**Figure 4:**
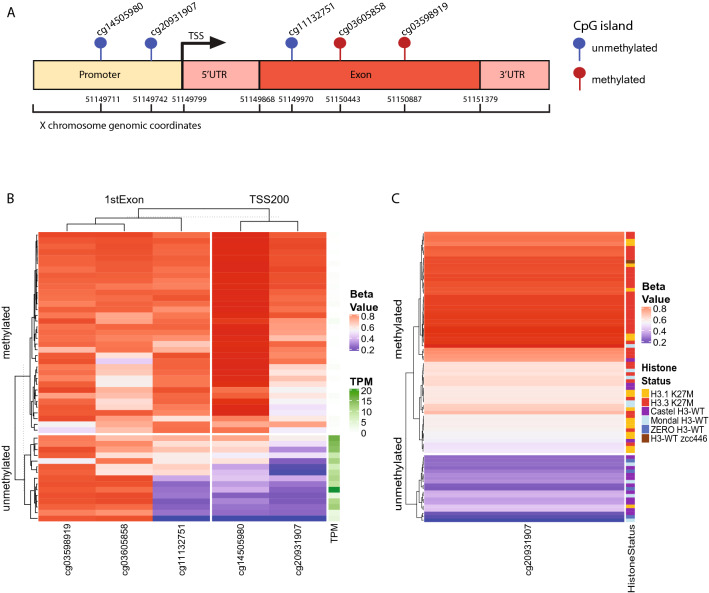
EZHIP expression is regulated by promoter methylation. (**A**) Lollipop depiction of the location of CpG islands on the Illumina EPIC 850 K methylation array that are located in either the 1st Exon or within 200 base pairs of the transcription start site (TSS200) of the *EZHIP* gene. The genomic coordinates are genome build hg19. CpG islands identified as unmethylated are blue and methylated red in H3-WT cohort. (**B**) Heatmap representation of the 5 CpG islands in *EZHIP* and the corresponding beta value for each ZERO glioma tumour sample and the TPM value (0 – white to 20 – dark green) for the corresponding sample. (**C**) Heatmap of the most correlated CpG island for ZERO, Mondal and Castel patients, with the histone status of each patient highlighted (H3.1 K27M – yellow; H3.3 K27M – red; Castel H3-WT – purple; Mondal H3-WT – light blue; ZERO H3-WT – blue; H3-WT zcc446 – brown). For B and C the beta value is a range between 0–1 with 0 (blue) being unmethylated, 1 (red) methylated and 0.5 (white).

*EZHIP* has recently been identified as an oncogenic driver in PFA-EPD^13^ and its overexpression has been previously reported to be associated with relative hypomethylation of its promoter compared to other subgroups of ependymomas^11^. We explored the potential mechanism regulating *EZHIP* expression by integrating the RNA-seq data with the methylation status of the CpG island probes at the *EZHIP* promoter within the ZERO cohort. On the Illumina EPIC 850K array, 5 probes are within a CpG island promoter region*,* located either within the 1st exon or 200 base pairs of the transcription start site (TSS200) (Fig. 4A). We showed a correlation between the DNA methylation status of the probes within the TSS200 and *EZHIP* expression as measured by transcripts per million (TPM) (Fig. 4B, Supplementary Fig. 3a). In all tumours where *EZHIP* was expressed, the probe cg20931907 (chrX:51149742), and to a lesser extent probes cg11132751 and cg14505980, were unmethylated, in keeping with the hypothesis that the methylation of CpG islands within the *EZHIP* promoter represses gene expression. To validate these findings, we examined the samples from the Castel cohort with reported elevated *EZHIP* expression. These samples also showed the same unmethylated CpG island profile (Fig. 4C, Supplementary Fig. 3b). We predict that samples from the Mondal cohort which clustered with the *EZHIP-*high H3-WT (but did not have RNA-seq or IHC data to confirm high *EZHIP* expression) would also be hypomethylated at these loci (Fig. 4C, Supplementary Fig. 3b). In keeping with these findings, we observed methylation of the *EZHIP* promoter in tumours not expressing *EZHIP,* including histone H3.3 K27M and H3.1 K27M DMGs. This was also observed in H3-WT sample zcc446 (lacking *EZHIP expression*), and interestingly there was another H3-WT sample in the Mondal cohort that exhibited a similar aneuploid profile as zcc446 with *EZHIP* promoter methylation. We conclude that *EZHIP* expression may be directly regulated by the methylation status of its promoter in gliomas.

### Global DNA methylation changes in H3-WT DMGs identifies DNA hypermethylation signatures correlated with the disruption of neurodevelopmental events

**Figure 5:**
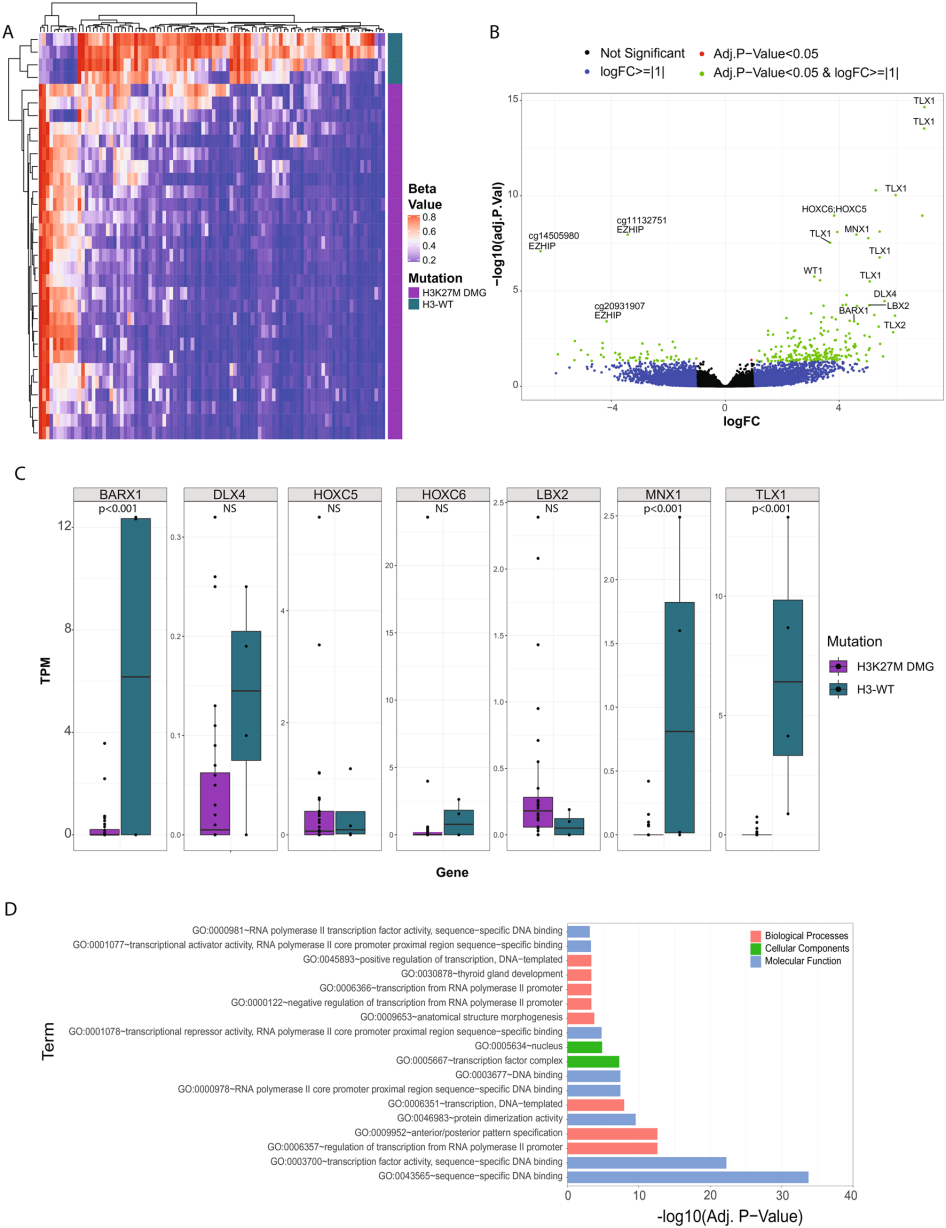
DMG and H3-WT patients have different methylation patterns of homeobox genes. (**A**) Heatmap representation of the significant differentially methylated probes comparing H3-WT (blue; N = 4) and H3K27M DMG (purple; N = 28). The beta value is a range between 0–1 with 0 (blue) being unmethylated, 1 (red) methylated and 0.5 (white). (**B**) Volcano plot of the differentially methylated probes comparing H3-WT midline gliomas and H3K27M DMG samples, where the x-axis is the log_2_-fold change (logFC) and y-axis the adjusted p-value (log_10_ scaled). Probes are highlighted as not significant (black), logFC > =|1| (blue), adjusted p-value < 0.05 (red) and both logFC > =|1| and adjusted p-value < 0.05 (green). Genes with a positive fold-change are more methylated and a negative fold-change is more unmethylated in H3-WT compared to H3K27M DMG samples. The three significantly differentially methylated *EZHIP* probes (unmethylated H3-WT) are labelled and significant homeobox family genes that were significantly differently methylated (methylated H3-WT) are also highlighted. (**C**) Relative TPM expression (y-axis) for each sample of the HOX family genes that were identified as differentially methylated between H3-WT (blue) and H3K27M DMG (purple) samples (NS = not significant) (**D**) GO enrichment analysis significant results from the significantly differentially methylated probes. The x-axis is the adjusted p-value (log_10_ scaled), and the y-axis is the GO term. GO enrichment terms are coloured according to; biological processes (pink), cellular components (green) and molecular function (blue).

We assessed the global DNA methylation differences between H3-WT and H3K27M DMGs from the ZERO cohort. This revealed that H3-WTs had a greater number of hypermethylated CpG sites than H3K27M DMGs (Fig. 5A, Supplementary Table 1). Strikingly though, in the H3-WT cohort, *EZHIP* was the only significantly unmethylated gene across the two groups (Fig. 5A,B). Several other differentially hypermethylated genes in the H3-WT group included homeobox family transcription factors *TLX1*, *HOXC5;HOXC6*, *MNX1*, *DLX4*, *LBX2,* and *BARX1* (Supplementary Table 1)*.* Many of these homeobox family of transcription factors are involved in neural differentiation and specification^31^*. WT1,* a key developmental gene^32^ playing a role in the regulation of DNA methylation in other types of cancer^33,34^ was found to be significantly hypermethylated as well (Fig. 5A,B). However, we did not see an inverse RNA expression pattern with the differentially methylated *HOX* genes between the H3-WT tumours and H3K27M DMGs (HOX expression < 15 TPM for all samples) (Fig. 5C). A GO enrichment analysis was conducted on the significant differentially methylated genes identified when comparing CpG island methylation status between H3-WT and H3K27M DMG samples. The analysis revealed an enrichment for transcription factor activity DNA binding, anatomical structure morphogenesis, sub-localisation to the nucleus and anterior/posterior specification ontologies (Fig. 5D), suggesting that H3-WT tumours may have a distinct cell of origin from H3K27M DMGs. Together our data shows that *EZHIP*-high H3-WT tumours are characterised by a global gain of DNA methylation of genes involved in normal development and morphogenesis.

## Discussion

Using a multi-platform molecular profiling approach (WGS, RNA-seq and methylation), we have further characterised genomic and clinical characteristics of the recently identified sub-group of DMGs with concurrent wild-type histone H3 genes, loss of H3K27me3 and *EZHIP* overexpression^6–9^. Our data suggests that the elevated *EZHIP,* a feature in this group of DMGs, may be explained by promotor hypomethylation. Our integrated approach confirmed the absence of mutations in epigenetic related genes. *ACVR1* and *EGFR* mutations were mutually exclusive with tumours clustering distinctly by both RNA expression and DNA methylation t-SNE plots, suggesting even further sub-classification within this subgroup. Moreover, our data supports the view that these tumours belong within the DMG grouping^4,6,7^ as has recently been recognised through the incorporation of the modified *DMG, H3-K27 altered* group in the 5^th^ edition of the WHO Classification of Tumours of the CNS^14^. As previously reported^17^, we confirmed that this subgroup could be diagnosed in a clinical setting by a specific immunohistochemistry profile combining loss of H3K27me3, negative H3-K27M, and positive nuclear staining of EZHIP antibody. Tumour zcc446 had a different molecular profile with aneuploid genome, lack of *AVCR1* or *EGFR* mutations and negative EZHIP IHC. This could be indicative of an additional uncharacterised *DMG, H3-K27 altered* subgroup.

Most DMGs are characterised by recurrent K27M alterations in H3.1 or H3.3 and both subtypes are characterised by a global loss of H3K27me3^1,35^. The mutated histones, either H3.1 or H3.3, delineate DMG sub-groups with different age of onset, location and co-occurring mutations^30^. These two groups also present different DNA methylation and gene expression profiles, suggesting that they may arise from a different precursor cell or that they may undergo alternate differentiation paths from the original precursor cell^36,37^. Our data further deepens the knowledge of the recently described subgroup of H3K27 altered DMG with loss of histone H3K27me3 and without a mutation in the histone genes, by adding our cohort to previously published cases. Concurrent with H3K27me3 loss, *EZHIP* is highly expressed in these tumours. *EZHIP* is a newly characterised gene initially described in PFA-EPD, where it mimics H3K27M mutant action through PRC2 inhibition leading to H3K27me3 loss^13^. *EZHIP* appears to have a similar oncogenic role in these DMGs, acting as a H3K27M mimic. In PFA-EPD, the level of *EZHIP* expression has been reported to correlate with the methylation status of its promoter^11^. We describe for the first time in H3-WT DMG similar findings, and we further hypothesise that dysregulation of *EZHIP* via promoter demethylation could be occurring as an early event in these tumours. Although we report a trend for H3-WT *ACVR1-*mutant tumours to express higher levels of *EZHIP*, our cohort size is too small for this observation to reach statistical significance and a larger cohort would be required to confirm our findings. Based on our results which associate *EZHIP* overexpression with its promoter demethylation, it would be interesting to see whether various levels of *EZHIP* expression correlate with a specific pattern of DNA methylation across the gene promoter but also the gene body and to further investigate what are the events leading to promoter demethylation.

Our observation that a subset of homeobox genes are methylated in H3-WT compared to H3K27M DMGs, is consistent with previous reports of unmethylated homeobox genes in H3K27M DMGs, providing further insight into the developmental nature of this tumour subset^38^. Homeobox genes play critical roles in normal brain development, and when dysregulated, drive oncogenic transformation. Several mechanisms of dysregulation of homeobox genes have been reported, including epigenetic alterations through DNA methylation changes at regulatory regions^39^. Considering that DNA methylation differences between H3K27M DMGs and H3-WT tumours involve homeobox genes with functions related to regulation and development suggests that H3K27M DMGs and H3-WT tumours may arise from different cell lineages or through blocks of alternate differentiation pathways. Interestingly, dysregulation of homeobox genes have previously been described in adult GBMs^40^, and more recently in H3.3 G34-mutant gliomas where dysregulated high expression of *GSX2* and *DLX1/2* reflects the stalled development of the tumour in an interneuron progenitor state^41^. Studies have demonstrated a strong preference of PRC2 binding and repression of genes involved in regulation of development and cell fate, including *HOX* and *TLX* genes^42–44^. *EZHIP* binds to the active site of the SET domain of EZH2 within the PRC2 complex^12^, thus, it is plausible that within the H3-WT cohort, binding of *EZHIP* to the PRC2 complex leads to differential DNA methylation of the homeobox genes observed. Studies by Nagaraja et al. and Castel et al. have found that the type of histone H3 mutation influences the deposition of the H3K27me3 marks and leads to a differing transcriptome and methylome profile^36,37^. It is also known that H3.1 K27M and H3.3 K27M are expressed at different phases of the cell cycle and stages of cellular differentiation^45,46^. Therefore, it is likely that *EZHIP* may have differential temporal expression and interacting partners from both histone variants leading to potential spatial changes in H3K27me3 marks and overall changes in the methylome profiles.

In this study, differential expression of the homeobox genes was not observed between the H3-WT and DMG groups, without any correlation observed with DNA methylation results. However, it should be noted that methylation was only studied at CpG islands, whilst it is known that CpG sites along the body of a gene can also alter gene expression^47^. Due to the complex interplay between DNA methylation, histone methylation, chromatin accessibility, *cis*-regulatory elements and regulation by non-coding RNAs, it is challenging to link specific molecular factors to HOX gene dysregulation^48,49^. A recent study conducting a comprehensive analysis of epigenetic changes and RNA expression at HOX clusters in adult gliomas highlighted this complex regulation where DNA hypermethylation and gene overexpression can coexist^49^, in keeping with our findings. Furthermore, based on chromatin immunoprecipitation sequencing data, Le Boiteux et al. suggested that HOX clusters dysregulation could be due to the use of an alternative TSS^49^. Unfortunately, our DNA methylation analysis was restricted to allow integration of cases with data publicly available, and therefore does not take into consideration alternative CpG sites or regulatory elements across the body that may be more representative of gene expression, and further analysis beyond the scope of this paper is warranted to understand the differential role of HOX genes in H3-WT tumours and DMGs.

Despite decades of collaborative efforts, the prognosis of patients with H3K27M DMG remains dismal. We report here that children affected with a H3-WT tumour with loss of H3K27me3 have the same poor prognosis and it is fitting that they are now considered a subset of the same disease entity. Therefore, these H3-WT tumours should be considered as HGGs irrespective of their histological grade. It is worth emphasising the importance of a layered diagnosis which should include characterisation of H3K27M, H3 K27me3 and *EZHIP* status. Moreover, patients with H3-WT tumours have distinct clinical features, with for example, tumours with *ACVR1* mutations occurring at a much older age than those associated with H3.1 K27M mutations. Furthermore, identification of novel potential therapeutic avenues is critical. Recently, *EZHIP* has been described as being involved in the homologous recombination repair pathway by displacing the *PALB2-BRCA2* interaction, leading to in vitro and in vivo activity of PARP inhibitors against PFA-EPD cells^50^. Alternatively, the use of a histone demethylase inhibitor can restore the epigenetic dynamics in the cell leading to increased survival in vivo^51,52^. Such an approach is currently being investigated in PFA-EPD and could be expanded to future investigation in H3-WT tumours (https://clinicaltrials.gov/ct2/show/NCT03206021). We also identify targetable oncogenic mutations already explored in H3K27M DMGs such as *ACVR1* or *PIK3CA.* A study has shown that inhibition of ALK2 encoded by *ACVR1* in H3K27M DMGs by pyrazolo[1,5-a]pyrimidine- and pyridine-based inhibitors prolonged survival of orthotopic mice^53,54^. Whereas inhibitors of the PI3K/mTOR pathway have been investigated in early phase trials with mixed results^55,56^. *EGFR* mutation is another targetable alteration identified in these tumours. Mondal et al. have previously reported that response to tyrosine kinase inhibitors (TKI) is dependent on the type of alteration and on the drug tested, and importantly patients treated with either a TKI or MEK inhibitor have a prolonged survival compared to their counterparts^7^. Further research is needed to identify synthetic lethal vulnerabilities in a tumour type where conventional approaches have been proven to be of limited benefit.

Our study reveals the unique molecular alterations underpinning H3-WT with H3K27me3 loss, provides molecular insight into *EZHIP* dysregulation and demonstrates the importance of sub-group classification and refinement. Our study emphasises the importance of comprehensive molecular profiling of these rare tumours given the dismal outcome and highlights the potential for personalised therapy recommendations which can hopefully lead to improved survival outcomes.

## Supplementary Information

Supplementary Information.

## Data Availability

This data is available by request through the ZERO childhood cancer data access committee and approved by the ZERO Research Management Committee by emailing zero@ccia.org.au.

## Abbreviations

ACVR1: Activin A receptor type 1
CNS: Central nervous system
DMG: Diffuse midline gliomas
EGFR: Epidermal growth factor receptor
EZHIP: EZH inhibitory protein
FFPE: Formalin-fixed paraffin embedded
GO: Gene ontology
GBM: Glioblastomas
H3K27me3: H3K27 trimethylation
H3-WT: Midline gliomas with wild-type histone H3 and H3K27me3 loss
HGG: High grade gliomas
IHC: Immunohistochemistry
PFA-EPD: Posterior fossa group A ependymomas
RNA-seq: RNA-sequencing
TPM: Transcripts per million
t-SNE: T-Distributed stochastic neighbour embedding
TSS200: 200 Base pairs of the transcription start site
WGS: Whole genome sequencing
ZERO: Zero childhood cancer precision medicine program
