# Supplementary material for: Histone H3-wild type diffuse midline gliomas with H3K27me3 loss are a distinct entity with exclusive EGFR or ACVR1 mutation and differential methylation of homeobox genes
Source: Sci Rep. 2023 Mar 7;13:3775. doi: 10.1038/s41598-023-30395-4 (PMC9992705; doi:10.1038/s41598-023-30395-4)
Supplement: Supplementary file 1 — Supplementary Information. [file 41598_2023_30395_MOESM1_ESM.pdf]

Histone H3-wild type diffuse midline gliomas with H3K27me3 loss are a distinct entity with exclusive *EGFR* or *ACVR1* mutation and differential methylation of homeobox genes

Pamela Ajuyah[1^], Chelsea Mayoh[1,2,3^], Loretta M.S. Lau[1,2,3,4], Paulette Barahona[1], Marie Wong[1,2,3], Hazel Chambers[5], Fatima Valdes-Mora[1,2], Akanksha Senapati[4], Andrew J. Gifford[1,2,6], Colleen D'Arcy[5], Jordan R. Hansford[7,8,9,10,11,12,13], Neevika Manoharan[2,4], Wayne Nicholls[14], Molly M. Williams[7], Paul J. Wood[15], Mark J. Cowley[1,2,3], Vanessa Tyrrell[1,2], Michelle Haber[1,2,3], Paul G. Ekert[1,2,3,16,17], David S. Ziegler[1,2,3,4*^], Dong-Anh Khuong-Quang[7,8*^]

[1]Children's Cancer Institute, Lowy Cancer Research Centre, UNSW Sydney, Kensington, NSW, Australia,
[2]School of Clinical Medicine, UNSW Medicine & Health, UNSW Sydney, Kensington, NSW, Australia
[3]University of New South Wales Centre for Childhood Cancer Research, UNSW, NSW, Australia
[4]Kids Cancer Centre, Sydney Children's Hospital, NSW, Australia
[5]Department of Anatomical Pathology, Royal Children's Hospital, University of Melbourne, Victoria, Australia
[6]Anatomical Pathology, NSW Health Pathology, Prince of Wales Hospital, Randwick, NSW, Australia
[7]Children's Cancer Centre, Royal Children's Hospital, Parkville, Victoria, Australia
[8]Murdoch Children's Research Institute, Royal Children's Hospital, Parkville, Victoria, Australia
[9]Department of Paediatrics, University of Melbourne, Parkville, Victoria, Australia
[10]Michael Rice Cancer Centre, Women's and Children's Hospital, Adelaide, South Australia, Australia
[11]South Australia Health and Medical Research Institute, Adelaide, South Australia, Australia
[12]South Australia Immunogenomics Cancer Institute, Adelaide, South Australia, Australia
[13]University of Adelaide, Adelaide, South Australia, Australia
[14]Oncology Service, Children's Health Queensland Hospital & Health Service, Brisbane, Queensland, Australia
[15]Department of Paediatrics, School of Clinical Sciences at Monash Health, Monash University, Clayton, Victoria, Australia
[16]Cancer Immunology Program, Peter MacCallum Cancer Centre, Parkville, Victoria, Australia
[17]The Sir Peter MacCallum Department of Oncology, University of Melbourne, Parkville, Victoria, Australia

^These authors contributed equally to this work

*Corresponding authors

Supplementary Table 1. CpG island probe positions of differentially methylated genes between H3-WT and H3K27M DMG tumours. List of probes altered in the differential methylation analysis between the H3-WT and H3K27M DMG tumours including chromosome positions, Gencode gene name, log fold change (FC), P-value, adjusted P-value and B-value.

Supplementary Fig. 1 MRI imaging and histological features of a 3 year-old male with H3-WT (zcc183) and EZHIP staining for zcc339 with positive control (a-b) Gadolinium-enhanced T1-weighted and T2-weighted axial images demonstrating the bilateral thalamic infiltrative H3-WT with scanty enhancement. (c) H&E and immunohistochemistry staining for zcc183 including H&E (600x), H3K27M (600x), H3K27me3 (600x) and EZHIP (600x), as well as EZHIP positive staining control (400x). Immunohistochemical staining demonstrates negative nuclear staining for H3K27M mutant, loss of nuclear staining for H3K27me3 in tumour cells with preserved staining in inflammatory cells, and strong nuclear expression of EZHIP in tumor cells.

Supplementary Fig. 2 Somatic mutation profile of 5 H3-WT tumours

Circos plots showing the somatic genome-wide mutation profile of **(a-b)** *ACVR1* mutated H3-WT tumours, **(c-d)** *EGFR* mutated H3-WT tumours and **(e)** H3-WT tumour. The inner area shows the structural variants, the inner ring shows the minor allele ploidy, followed by the copy number variants (green are amplifications, and red are deletions), then somatic SNVs (C>A – blue, C>G – black, C>T – red, T>A – grey, T>C – light green, T>G – light pink).

Supplementary Fig. 3 EZHIP expression is regulated by promoter methylation

Heatmap representation of the 5 CpG islands in *EZHIP.* **(a)** The ZERO glioma tumour cohort with Mondal and Castel cohorts with histology highlighted (H3K27M DMG – red; ependymoma (EPD) other – pink; PFA-EPD – purple; posterior fossa B (PFB-EPD) – dark green; RELA fusion positive EPD (RELA-EPD) – light green; H3-WT – blue; H3-WT zcc446 – brown; HGG – yellow; LGG – light blue) the TPM value (0 – white to 20 – dark green, grey – unknown TPM) for the corresponding sample (N=138) and **(b)** Mondal, Castel and ZERO H3-WT (purple, light blue and blue, respectively, and brown for zcc446 H3-WT sample without *EZHIP* expression) and H3K27M DMG (H3.1 K27M – yellow; H3.3 K27M – red) (N=82).

Supplementary Table 1. CpG island probe positions of differentially methylated genes between H3-WT and H3K27M DMG tumours.

Name	chr	pos	Islands_Name	Relation_to_Island	GencodeBasicV12_NAME	GencodeBasicV12_Group	logFC	AveExpr	t	P.Value	adj.P.Val	B
cg25266629	chr10	102894148	chr10:102893660-102895059	Island	TLX1;RP11-108L7.11	ExonBnd;3'UTR	6.9952	3.7689	21.7518	5.25E-21	2.24E-15	28.1803
cg24812837	chr10	102894120	chr10:102893660-102895059	Island	TLX1;RP11-108L7.11	ExonBnd;3'UTR	6.9845	4.0539	19.7173	1.04E-19	2.96E-14	26.7155
cg14861089	chr10	102895043	chr10:102893660-102895059	Island	RP11-108L7.11	3'UTR	5.2847	3.6469	14.8667	4.27E-16	5.19E-11	21.9102
cg01175020	chr10	102896475	chr10:102896342-102896665	Island	RP11-31L23.3;TLX1;RP11-108L7.11	3'UTR;3'UTR;3'UTR	5.9850	2.4836	14.4974	8.73E-16	9.31E-11	21.4476
cg07080050	chr12	54413345	chr12:54412990-54413346	Island	AC012531.25;HOXC6;HOXC4;HOXC5	TSS1500;5'UTR;5'UTR;5'UTR	3.8250	3.2596	13.0081	1.81E-14	1.10E-09	19.4102
cg05482942	chr10	102899285	chr10:102899177-102899489	Island	RP11-31L23.3;RP11-31L23.3;RP11-108L7.11	3'UTR;1stExon;3'UTR	6.9216	3.6918	12.9739	1.95E-14	1.11E-09	19.3601
cg07494667	chr10	102894547	chr10:102893660-102895059	Island	RP11-108L7.11	3'UTR	5.4199	3.5251	12.0312	1.51E-13	7.59E-09	17.9106
cg07915434	chr10	102894342	chr10:102893660-102895059	Island	RP11-108L7.11	3'UTR	3.9317	1.5579	11.9836	1.68E-13	7.97E-09	17.8340
cg11132751	chrX	51149970	chrX:51149661-51150911	Island	CXorf67	1stExon	-3.4214	-2.1387	-11.7684	2.73E-13	1.10E-08	17.4834
cg27651243	chr7	156799148	chr7:156795355-156799394	Island	RP5-1121A15.4;MNX1	5'UTR;3'UTR	4.6064	3.7913	11.7525	2.83E-13	1.10E-08	17.4572
cg07416656	chr10	102894441	chr10:102893660-102895059	Island	RP11-108L7.11	3'UTR	5.0219	2.9929	11.5470	4.52E-13	1.67E-08	17.1153
cg04552206	chr10	102896546	chr10:102896342-102896665	Island	RP11-31L23.3;TLX1;RP11-108L7.11	3'UTR;3'UTR;3'UTR	3.6854	2.0422	11.2892	8.18E-13	2.91E-08	16.6772
cg14505980	chrX	51149711	chrX:51149661-51150911	Island	CXorf67	TSS200	-6.4879	-4.4391	-10.8174	2.48E-12	8.13E-08	15.8484
cg06382559	chr10	102896640	chr10:102896342-102896665	Island	RP11-31L23.3;TLX1;RP11-108L7.11	3'UTR;3'UTR;3'UTR	5.4216	3.5741	10.4890	5.45E-12	1.72E-07	15.2503
cg19211915	chr11	32452513	chr11:32452144-32452708	Island	WT1	TSS200	3.1293	2.3425	9.4047	8.13E-11	1.75E-06	13.1483
cg04273871	chr10	102894227	chr10:102893660-102895059	Island	RP11-108L7.11	3'UTR	3.3276	1.4737	9.2098	1.34E-10	2.73E-06	12.7494
cg00741609	chr10	102893925	chr10:102893660-102895059	Island	TLX1;TLX1;RP11-108L7.11	5'UTR;ExonBnd;3'UTR	5.0745	2.3194	9.1386	1.62E-10	3.13E-06	12.6021
cg08487063	chr10	102894639	chr10:102893660-102895059	Island	RP11-108L7.11	3'UTR	4.2651	2.2586	8.4254	1.06E-09	1.65E-05	11.0790
cg01832036	chr17	48050274	chr17:48048953-48050616	Island	DLX4;DLX4	5'UTR;1stExon	5.5963	4.2170	8.1279	2.38E-09	3.39E-05	10.4183
cg14258623	chr6	1384591	chr6:1381743-1385211	Island	RP4-668J24.2	1stExon	4.2411	4.7073	7.9088	4.34E-09	5.22E-05	9.9226
cg03270710	chr2	74729592	chr2:74729399-74731166	Island	RP11-523H20.2;LBX2	TSS200;3'UTR	5.0556	2.4836	7.8695	4.84E-09	5.56E-05	9.8329
cg27521476	chr10	102896376	chr10:102896342-102896665	Island	RP11-31L23.3;RP11-108L7.11	3'UTR;3'UTR	4.1243	1.7150	7.8682	4.86E-09	5.56E-05	9.8298
cg08622757	chr7	156799393	chr7:156795355-156799394	Island	RP5-1121A15.4;MNX1	5'UTR;3'UTR	3.4509	3.0818	7.8198	5.55E-09	5.92E-05	9.7190
cg19164987	chr12	54413000	chr12:54412990-54413346	Island	AC012531.25;HOXC6;HOXC4;HOXC5	TSS1500;5'UTR;5'UTR;5'UTR	4.6247	3.4338	7.8029	5.82E-09	6.12E-05	9.6802
cg02167020	chr10	102899949	chr10:102899822-102900263	Island	RP11-31L23.3;RP11-108L7.11	TSS1500;3'UTR	3.0164	3.9318	7.4288	1.65E-08	0.00015	8.8098
cg15691862	chr1	47905438	chr1:47902793-47905518	Island	FOXD2;FOXD2	1stExon;3'UTR	5.2348	2.7909	7.3424	2.10E-08	0.00018	8.6059
cg08355260	chr2	74729429	chr2:74729399-74731166	Island	RP11-523H20.2;LBX2	TSS1500;3'UTR	5.9597	2.2252	7.3073	2.32E-08	0.0002	8.5226
cg14383658	chr10	102899262	chr10:102899177-102899489	Island	RP11-31L23.3;RP11-31L23.3;RP11-108L7.11	3'UTR;1stExon;3'UTR	3.7445	2.4598	7.2278	2.91E-08	0.00024	8.3335
cg10738368	chr2	74729551	chr2:74729399-74731166	Island	RP11-523H20.2;LBX2	TSS200;3'UTR	3.4607	1.7332	7.2169	3.00E-08	0.00024	8.3076
cg03186004	chr1	6480288	chr1:6479057-6480938	Island	HES2;HES2;HES2;HES2	5'UTR;TSS1500;TSS1500;TSS1500	2.1473	2.8525	7.1822	3.31E-08	0.00026	8.2246
cg02440420	chr7	156798931	chr7:156795355-156799394	Island	RP5-1121A15.4;MNX1	TSS200;3'UTR	3.8796	4.9097	7.1263	3.88E-08	0.0003	8.0907
cg15689410	chr2	74727000	chr2:74725039-74727038	Island	LBX2;LBX2;AC005041.17	TSS200;3'UTR;TSS1500	4.3670	3.6799	7.0542	4.76E-08	0.00036	7.9173
cg20931907	chrX	51149742	chrX:51149661-51150911	Island	CXorf67	TSS200	-4.1678	-1.7291	-7.0270	5.14E-08	0.00039	7.8518
cg13773631	chr9	96715843	chr9:96713326-96718186	Island	BARX1	TSS200	4.5243	2.4115	7.0178	5.28E-08	0.00039	7.8296
cg09136529	chr9	96715872	chr9:96713326-96718186	Island	BARX1	TSS200	3.3656	1.9185	6.9811	5.86E-08	0.00043	7.7408
cg14487292	chr17	36105517	chr17:36105334-36105583	Island	RP11-115K3.1;HNF1B;HNF1B;HNF1B	5'UTR;TSS1500;TSS1500;TSS1500	4.6525	2.8122	6.8834	7.75E-08	0.00052	7.5038
cg01295339	chr2	74729710	chr2:74729399-74731166	Island	RP11-523H20.2;LBX2	TSS200;3'UTR	5.3886	2.8529	6.7350	1.19E-07	0.00074	7.1415
cg07209634	chr7	156796550	chr7:156795355-156799394	Island	MNX1	3'UTR	3.3599	2.1939	6.6707	1.43E-07	0.00088	6.9836
cg00927495	chr9	96715687	chr9:96713326-96718186	Island	BARX1;BARX1	1stExon;5'UTR	3.9379	2.4815	6.5789	1.86E-07	0.00108	6.7573
cg12251336	chr16	216719	chr16:214343-216720	Island	HBM;HBM	3'UTR;5'UTR	2.4579	2.0809	6.5372	2.10E-07	0.00117	6.6542
cg16424078	chr6	1384705	chr6:1381743-1385211	Island	RP4-668J24.2	1stExon	3.4671	3.6756	6.5217	2.20E-07	0.00121	6.6159
cg03848675	chr6	1389146	chr6:1389139-1391393	Island	FOXF2	TSS1500	1.5038	4.7328	6.5126	2.25E-07	0.00123	6.5933
cg13491462	chr6	1384491	chr6:1381743-1385211	Island	RP4-668J24.2	1stExon	2.4210	4.1439	6.5038	2.31E-07	0.00125	6.5716
cg09234616	chr11	32452592	chr11:32452144-32452708	Island	WT1	TSS1500	3.6097	3.3477	6.4815	2.47E-07	0.00131	6.5161
cg21185289	chr2	74743437	chr2:74740455-74743795	Island	TLX2	3'UTR	5.8979	4.1817	6.4331	2.84E-07	0.00147	6.3960
cg20912770	chr11	2292428	chr11:2290104-2292932	Island	ASCL2	TSS1500	2.1901	3.9006	6.4192	2.96E-07	0.0015	6.3613
cg19500479	chr2	20865674	chr2:20865289-20867589	Island	GDF7	TSS1500	3.3461	2.9450	6.3762	3.35E-07	0.00167	6.2543
cg04255230	chr2	74727010	chr2:74725039-74727038	Island	LBX2;LBX2;AC005041.17	TSS200;3'UTR;TSS1500	4.3518	2.9581	6.3657	3.45E-07	0.00169	6.2280
cg24625136	chr3	138658554	chr3:138656627-138659107	Island	RP11-548O1.3	3'UTR	1.6784	3.8652	6.3120	4.04E-07	0.00193	6.0939
cg21098990	chr19	10399845	chr19:10399844-10405375	Island	ICAM5	TSS1500	1.5022	5.1775	6.2992	4.19E-07	0.00197	6.0620
cg13854296	chr9	96716209	chr9:96713326-96718186	Island	BARX1	TSS1500	3.4055	1.2941	6.2805	4.42E-07	0.00203	6.0150
cg01693350	chr11	32452187	chr11:32452144-32452708	Island	WT1;WT1	1stExon;5'UTR	4.8911	1.7987	6.2488	4.85E-07	0.00215	5.9358
cg06268694	chr22	46932642	chr22:46929317-46934861	Island	CELSR1	1stExon	4.2378	3.1586	6.2297	5.13E-07	0.00225	5.8878
cg02893180	chr22	50919493	chr22:50919453-50920376	Island	ADM2;ADM2;ADM2	TSS1500;TSS1500;TSS1500	5.0676	3.4651	6.1665	6.17E-07	0.00252	5.7291
cg09839170	chr5	134376247	chr5:134374385-134376751	Island	CTC-276P9.1;CTC-276P9.1;CTC-203F4.1;CTC-349C3.1;CTC-349C3.1	TSS1500;TSS1500;5'UTR;5'UTR;5'UTR	2.3970	2.6275	6.1598	6.29E-07	0.00254	5.7122
cg02511456	chr7	156801419	chr7:156801418-156801632	Island	MNX1	3'UTR	2.2006	3.9531	6.0768	8.01E-07	0.00293	5.5033
cg07076509	chr9	96720819	chr9:96720586-96723189	Island	RP11-231K24.2	5'UTR	4.3826	2.2175	6.0561	8.51E-07	0.00305	5.4510
cg15446391	chr11	32452370	chr11:32452144-32452708	Island	WT1	TSS200	2.6090	0.7892	6.0507	8.65E-07	0.00305	5.4374
cg08490768	chr2	74730047	chr2:74729399-74731166	Island	RP11-523H20.2;RP11-523H20.2;LBX2;LBX2;LBX2;LBX2;LBX2	1stExon;5'UTR;1stExon;1stExon;1stExon;5'UTR;5'UTR;3'UTR	2.3202	2.1902	6.0412	8.89E-07	0.00308	5.4133
cg10748765	chr20	3218905	chr20:3218578-3220930	Island	SLC4A11;SLC4A11;SLC4A11	TSS1500;TSS200;3'UTR	3.1319	3.5696	6.0381	8.97E-07	0.00308	5.4055
cg14101207	chr17	48050381	chr17:48048953-48050616	Island	DLX4	1stExon	2.9726	2.8346	5.9600	1.13E-06	0.0037	5.2080
cg13230606	chr17	36105337	chr17:36105334-36105583	Island	RP11-115K3.1;RP11-115K3.1;HNF1B;HNF1B	1stExon;5'UTR;TSS1500;TSS200;TSS1500	3.8818	3.2234	5.9479	1.17E-06	0.00376	5.1773
cg03935183	chr10	8100563	chr10:8100384-8100768	Island	GATA3;GATA3	1stExon;5'UTR	3.8508	-0.1943	5.9407	1.19E-06	0.00382	5.1591
cg24426072	chr7	28997173	chr7:28995305-28998541	Island	TRIL;AC005013.5;AC005013.5	1stExon;5'UTR;TSS200	-5.2829	0.5031	-5.8947	1.36E-06	0.0042	5.0426
cg02272993	chr2	45159972	chr2:45159893-45160112	Island	RP11-89K21.1;RP11-89K21.1;RP11-89K21.1;RP11-89K21.1	3'UTR;3'UTR;3'UTR;3'UTR	4.2174	2.6228	5.8670	1.48E-06	0.00439	4.9722
cg04876028	chr10	134937967	chr10:134937966-134938481	Island	GPR123	3'UTR	-1.8419	-1.5710	-5.8434	1.59E-06	0.00458	4.9122

cg06005891	chr6	1389167	chr6:1389139-1391393	Island	FOXF2	TSS1500	2.6530	3.9643	5.8343	1.63E-06	0.00468	4.8891
cg13112154	chr10	102996565	chr10:102996034-102996646	Island	RP11-107I14.1;RP11-107I14.1	1stExon;5'UTR	4.2275	3.0533	5.8132	1.73E-06	0.00489	4.8355
cg07679948	chr12	56329641	chr12:56329518-56329790	Island	DGKA;DGKA;DGKA;DGKA	5'UTR;5'UTR;5'UTR;5'UTR	-4.3077	-2.2518	-5.7747	1.94E-06	0.0053	4.7375
cg09619786	chr15	75249914	chr15:75248277-75249922	Island	RPP25;RPP25	TSS1500;TSS200	-2.9112	1.8522	-5.7320	2.20E-06	0.0057	4.6288
cg17305266	chr6	1391265	chr6:1389139-1391393	Island	FOXF2	1stExon	2.5977	4.1902	5.7323	2.20E-06	0.0057	4.6295
cg12179826	chr9	96716020	chr9:96713326-96718186	Island	BARX1	TSS1500	5.4388	2.9515	5.7094	2.35E-06	0.00596	4.5711
cg14673618	chr2	45170322	chr2:45169505-45171884	Island	AC012354.5	TSS1500	4.5537	1.6658	5.7013	2.41E-06	0.00605	4.5505
cg10782923	chr12	56329731	chr12:56329518-56329790	Island	DGKA;DGKA;DGKA;DGKA	5'UTR;5'UTR;5'UTR;5'UTR	-2.3136	-1.6180	-5.6733	2.61E-06	0.00644	4.4791
cg10855681	chr2	74729476	chr2:74729399-74731166	Island	RP11-523H20.2;LBX2	TSS1500;3'UTR	3.4698	2.8484	5.6663	2.67E-06	0.0065	4.4611
cg00567703	chr12	54413101	chr12:54412990-54413346	Island	AC012531.25;HOXC6;HOXC4;HOXC5	TSS1500;5'UTR;5'UTR;5'UTR	2.9851	1.5597	5.6474	2.82E-06	0.00675	4.4129
cg16076038	chr22	19744078	chr22:19742901-19744729	Island	TBX1	TSS200	2.4613	0.6721	5.6263	3.00E-06	0.00701	4.3591
cg17421991	chr7	156795356	chr7:156795355-156799394	Island	MNX1	3'UTR	2.5685	3.6008	5.6080	3.17E-06	0.00727	4.3124
cg13460858	chr9	96720652	chr9:96720586-96723189	Island	RP11-231K24.2	5'UTR	3.0328	0.5867	5.6036	3.21E-06	0.00731	4.3011
cg11985360	chr22	19138209	chr22:19136293-19138512	Island	GSC2	TSS1500	2.2415	4.4527	5.5669	3.57E-06	0.00779	4.2071
cg15487105	chr22	19744051	chr22:19742901-19744729	Island	TBX1	TSS200	4.5010	2.4462	5.5661	3.58E-06	0.00779	4.2051
cg00884093	chr22	46932648	chr22:46929317-46934861	Island	CELSR1	1stExon	4.5790	4.5084	5.5605	3.64E-06	0.00782	4.1909
cg14097304	chr7	156801587	chr7:156801418-156801632	Island	MNX1	3'UTR	1.9712	2.7397	5.5393	3.88E-06	0.0082	4.1366
cg14566738	chr1	47905506	chr1:47902793-47905518	Island	FOXD2;FOXD2	1stExon;3'UTR	1.7774	1.1450	5.5306	3.98E-06	0.00838	4.1143
cg06768010	chr2	74729502	chr2:74729399-74731166	Island	RP11-523H20.2;LBX2	TSS1500;3'UTR	2.7891	1.1179	5.5193	4.11E-06	0.00854	4.0854
cg26746540	chr5	134375935	chr5:134374385-134376751	Island	CTC-276P9.1;CTC-276P9.1;CTC-203F4.1;CTC-349C3.1;CTC-349C3.1	TSS1500;TSS200;5'UTR;5'UTR;5'UTR	3.0252	2.9876	5.5188	4.12E-06	0.00854	4.0843
cg18595867	chr1	47899212	chr1:47899125-47899398	Island	RP11-511I2.5	1stExon	2.5410	1.3945	5.5085	4.24E-06	0.00871	4.0578
cg18250846	chr2	119592603	chr2:119592602-119593845	Island	RP11-19E11.1;RP11-19E11.1	5'UTR;1stExon	4.2378	1.7074	5.4911	4.47E-06	0.00906	4.0131
cg16076997	chr1	47905067	chr1:47902793-47905518	Island	FOXD2	1stExon	2.7459	2.6729	5.4856	4.54E-06	0.0091	3.9992
cg01060471	chr10	103911733	chr10:103911696-103912546	Island	NOLC1;NOLC1;NOLC1	TSS1500;TSS1500;TSS200	-1.7638	1.9891	-5.4806	4.61E-06	0.0092	3.9863
cg00462107	chr5	72677230	chr5:72676120-72678421	Island	RP11-79P5.8	TSS1500	4.5358	2.0083	5.4724	4.72E-06	0.00936	3.9653
cg02850487	chr17	27044852	chr17:27044168-27045049	Island	RAB34;RAB34;RAB34;RAB34;RAB34;RAB34;RAB34	1stExon;1stExon;5'UTR;5'UTR;5'UTR;5'UTR;TSS200	-4.3070	0.7413	-5.4351	5.26E-06	0.00993	3.8698
cg08428129	chr22	19744239	chr22:19742901-19744729	Island	TBX1;TBX1	1stExon;5'UTR	5.0691	3.1572	5.4289	5.36E-06	0.01004	3.8540
cg00598429	chr17	45928250	chr17:45928211-45928710	Island	SP6;SP6	5'UTR;5'UTR	3.6554	3.2316	5.4251	5.42E-06	0.01011	3.8442
cg11051055	chr8	11058145	chr8:11057684-11058479	Island	XKR6;XKR6	1stExon;1stExon	2.8227	4.5228	5.4018	5.81E-06	0.01051	3.7845
cg15409013	chr5	5139855	chr5:5139441-5140941	Island	CTD-2297D10.2;ADAMTS16	3'UTR;TSS1500	3.2277	3.7770	5.3816	6.16E-06	0.01087	3.7327
cg06071058	chr9	96716091	chr9:96713326-96718186	Island	BARX1	TSS1500	3.7599	1.0744	5.3541	6.68E-06	0.01137	3.6621
cg15019790	chr2	45236267	chr2:45235511-45237792	Island	SIX2;SIX2	5'UTR;1stExon	3.5569	3.0434	5.3461	6.84E-06	0.01159	3.6415
cg14994060	chr5	134376489	chr5:134374385-134376751	Island	CTC-276P9.1;CTC-276P9.1;CTC-203F4.1;CTC-349C3.1;CTC-349C3.1	TSS1500;TSS1500;5'UTR;5'UTR;5'UTR	1.6147	2.8138	5.3453	6.86E-06	0.01159	3.6396
cg08296831	chr11	65554175	chr11:65553749-65555573	Island	OVOL1	TSS1500	4.0504	4.2025	5.3414	6.93E-06	0.01163	3.6295
cg13409449	chr10	8097354	chr10:8091374-8098329	Island	GATA3;GATA3;GATA3	5'UTR;5'UTR;5'UTR	4.3470	3.3020	5.3313	7.14E-06	0.01175	3.6037
cg02887841	chr22	19744670	chr22:19742901-19744729	Island	TBX1	5'UTR	5.0425	3.5857	5.3105	7.59E-06	0.01234	3.5503
cg08513472	chr6	1625238	chr6:1624185-1625468	Island	GMDS	3'UTR	-5.0642	1.2635	-5.2985	7.87E-06	0.01261	3.5194
cg25010880	chr12	49351320	chr12:49350839-49351387	Island	ARF3;ARF3;ARF3;ARF3;AC073610.1;ARF3;ARF3	1stExon;TSS200;TSS1500;TSS200;TSS200;TSS200;5'UTR	-2.2275	2.6889	-5.2987	7.86E-06	0.01261	3.5200
cg13457209	chr10	28035129	chr10:28030182-28035211	Island	MKX;MKX;RP11-360I20.2	TSS1500;TSS200;5'UTR	2.4141	3.8016	5.2827	8.24E-06	0.01299	3.4789
cg26702958	chr10	102996254	chr10:102996034-102996646	Island	RP11-107I14.1;RP11-107I14.1	1stExon;5'UTR	4.2855	2.8069	5.2810	8.28E-06	0.013	3.4746
cg21174533	chr14	102026555	chr14:102025989-102031567	Island	DIO3OS;DIO3OS;DIO3OS;DIO3OS;DIO3OS;DIO3OS;DIO3OS;DIO3OS;DIO3OS;DIO3;DI	3'UTR;1stExon;3'UTR;3'UTR;3'UTR;3'UTR;TSS1500;3'UTR;1stExon;TSS1500;TSS1	3.5288	5.0298	5.2680	8.61E-06	0.01331	3.4411
cg01565438	chr17	33776554	chr17:33776553-33776888	Island	SLFN13;SLFN13;SLFN13;SLFN13	TSS1500;TSS1500;TSS1500;TSS1500	2.4824	1.2477	5.2601	8.81E-06	0.01346	3.4209
cg14823763	chr5	1884365	chr5:1881924-1887743	Island	IRX4;IRX4;IRX4;CTD-2194D22.3	5'UTR;5'UTR;TSS1500;5'UTR	3.1123	2.9847	5.2605	8.80E-06	0.01346	3.4220
cg12266953	chr2	20866261	chr2:20865289-20867589	Island	GDF7	TSS200	1.1923	5.2713	5.2504	9.06E-06	0.01366	3.3961
cg23237183	chr2	73429791	chr2:73429283-73430647	Island	NOTO;NOTO	5'UTR;1stExon	1.9475	3.8287	5.2136	1.01E-05	0.01471	3.3016
cg03895392	chr15	74426334	chr15:74425091-74428821	Island	ISLR2	5'UTR	3.1623	1.5655	5.1993	1.05E-05	0.01503	3.2649
cg05672223	chr2	43328033	chr2:43327936-43328337	Island	AC093609.1	3'UTR	-2.7393	2.1103	-5.1943	1.07E-05	0.01523	3.2519
cg10647101	chr22	19744026	chr22:19742901-19744729	Island	TBX1	TSS200	3.5212	2.1538	5.1931	1.07E-05	0.01526	3.2488
cg15724256	chr2	119602857	chr2:119602616-119604486	Island	EN1	TSS1500	3.6784	2.1995	5.1886	1.09E-05	0.01536	3.2374
cg16201038	chr13	28544760	chr13:28544524-28544890	Island	CDX2;CDX2	1stExon;5'UTR	3.7403	3.5113	5.1864	1.09E-05	0.01541	3.2317
cg14971597	chr6	159590578	chr6:159589636-159591319	Island	FNDC1;FNDC1	5'UTR;1stExon	2.4377	4.5621	5.1369	1.26E-05	0.01708	3.1046
cg04640971	chr2	45165318	chr2:45164561-45166567	Island	RP11-89K21.1;RP11-89K21.1	3'UTR;3'UTR	3.2732	2.0416	5.1331	1.28E-05	0.0171	3.0948
cg04840356	chr2	239755585	chr2:239755096-239758310	Island	TWIST2	TSS1500	3.4914	2.6576	5.1240	1.31E-05	0.01741	3.0715
cg04146591	chr17	27044864	chr17:27044168-27045049	Island	RAB34;RAB34;RAB34;RAB34;RAB34;RAB34;RAB34	1stExon;1stExon;5'UTR;5'UTR;5'UTR;5'UTR;TSS200	-3.8615	-0.0660	-5.1204	1.33E-05	0.01755	3.0622
cg18785598	chr7	156799021	chr7:156795355-156799394	Island	RP5-1121A15.4;RP5-1121A15.4;MNX1	1stExon;5'UTR;3'UTR	3.1908	2.0738	5.0870	1.46E-05	0.01866	2.9765
cg04486617	chr9	96715819	chr9:96713326-96718186	Island	BARX1	TSS200	2.8398	2.8722	5.0838	1.48E-05	0.01869	2.9683
cg14502484	chr5	1446208	chr5:1444678-1446648	Island	SLC6A3	TSS1500	3.5897	3.4172	5.0787	1.50E-05	0.01884	2.9550
cg05136263	chr2	45236019	chr2:45235511-45237792	Island	SIX2	1stExon	3.5538	1.1354	5.0703	1.54E-05	0.01906	2.9335
cg19470379	chr15	74425092	chr15:74425091-74428821	Island	ISLR2;ISLR2;ISLR2;ISLR2;ISLR2;ISLR2;ISLR2;ISLR2;ISLR2	5'UTR;5'UTR;5'UTR;5'UTR;5'UTR;5'UTR;5'UTR;ExonBnd;5'UTR	4.6174	1.9259	5.0721	1.53E-05	0.01906	2.9383
cg21109038	chr15	90543610	chr15:90543410-90543835	Island	ZNF710	TSS1500	-2.9598	1.3400	-5.0581	1.59E-05	0.0195	2.9022
cg14882669	chr7	28996420	chr7:28995305-28998541	Island	TRIL;AC005013.5;AC005013.5	1stExon;TSS1500;5'UTR	-3.7040	0.9584	-5.0553	1.61E-05	0.01956	2.8951
cg09658126	chr12	66524763	chr12:66524464-66524792	Island	RP11-745O10.2;LLPH;LLPH;RP11-745O10.4;TMBIM4	5'UTR;TSS1500;TSS1500;3'UTR;3'UTR	1.7817	4.9611	5.0407	1.68E-05	0.02017	2.8575
cg10895452	chr2	119602645	chr2:119602616-119604486	Island	EN1	TSS1500	2.3041	3.0851	5.0273	1.74E-05	0.02071	2.8232
cg14996783	chr7	28996902	chr7:28995305-28998541	Island	TRIL;AC005013.5;AC005013.5	1stExon;TSS1500;5'UTR	-5.8792	0.5676	-5.0221	1.77E-05	0.02095	2.8098
cg27091900	chr1	6484872	chr1:6484503-6485327	Island	HES2;ESPN;ESPN	TSS200;5'UTR;1stExon	2.6029	4.9521	5.0177	1.79E-05	0.02107	2.7984
cg08839210	chr17	27045048	chr17:27044168-27045049	Island	RAB34;RAB34;RAB34;RPL23A;RAB34;RAB34	5'UTR;5'UTR;TSS200;TSS1500;TSS200;TSS1500	-2.7160	0.6406	-5.0137	1.82E-05	0.02112	2.7882
cg15048991	chr5	5139866	chr5:5139441-5140941	Island	CTD-2297D10.2;ADAMTS16	3'UTR;TSS1500	3.7279	3.6779	5.0125	1.82E-05	0.02112	2.7851
cg10863075	chr20	5296267	chr20:5296266-5297798	Island	PROKR2;PROKR2	5'UTR;TSS1500	1.3889	2.8719	5.0116	1.83E-05	0.02113	2.7827

cg08800613	chr2	1595498	chr2:1595402-1596020	Island	AC144450.1	3'UTR	-3.7423	-0.8250	-5.0026	1.88E-05	0.02155	2.7598
cg10479082	chr7	28996639	chr7:28995305-28998541	Island	TRIL;AC005013.5;AC005013.5	1stExon;TSS1500;5'UTR	-4.8295	0.6378	-4.9869	1.96E-05	0.02212	2.7194
cg19504123	chr17	27044881	chr17:27044168-27045049	Island	RAB34;RAB34;RAB34;RAB34;RAB34;RAB34	1stExon;5'UTR;5'UTR;5'UTR;TSS200;TSS200	-4.3621	0.5397	-4.9803	2.00E-05	0.02234	2.7025
cg03377610	chr2	45164723	chr2:45164561-45166567	Island	RP11-89K21.1;RP11-89K21.1	3'UTR;3'UTR	2.9205	2.4417	4.9684	2.07E-05	0.02267	2.6719
cg04136610	chr5	5139878	chr5:5139441-5140941	Island	CTD-2297D10.2;ADAMTS16	3'UTR;TSS1500	3.5039	3.1490	4.9682	2.07E-05	0.02267	2.6715
cg06738242	chr2	239755703	chr2:239755096-239758310	Island	TWIST2	TSS1500	3.3629	2.1592	4.9705	2.06E-05	0.02267	2.6774
cg10954469	chr2	71115370	chr2:71114733-71115474	Island	AC007040.5	5'UTR	4.9457	2.8462	4.9648	2.10E-05	0.0227	2.6627
cg12318501	chr22	50919512	chr22:50919453-50920376	Island	ADM2;ADM2;ADM2	TSS1500;TSS1500;TSS1500	4.3394	2.2720	4.9638	2.10E-05	0.02271	2.6600
cg01499197	chr13	28544814	chr13:28544524-28544890	Island	CDX2;CDX2	1stExon;5'UTR	2.9557	1.8006	4.9579	2.14E-05	0.02281	2.6450
cg05106269	chr19	10381393	chr19:10380287-10381965	Island	ICAM1;ICAM1	TSS1500;TSS200	2.4946	2.1926	4.9581	2.14E-05	0.02281	2.6454
cg25222010	chr2	71017961	chr2:71017028-71017976	Island	FIGLA	TSS200	3.8729	1.0264	4.9595	2.13E-05	0.02281	2.6492
cg05309948	chr11	2292447	chr11:2290104-2292932	Island	ASCL2	TSS1500	2.2301	3.8243	4.9503	2.19E-05	0.0231	2.6255
cg09904891	chr22	19743689	chr22:19742901-19744729	Island	TBX1	TSS1500	3.1826	3.5814	4.9490	2.20E-05	0.02314	2.6221
cg02845591	chr2	74730523	chr2:74729399-74731166	Island	RP11-523H20.2;RP11-523H20.2;LBX2;LBX2;LBX2;LBX2	1stExon;5'UTR;1stExon;5'UTR;TSS1500;TSS200	1.3917	3.5588	4.9415	2.24E-05	0.0234	2.6028
cg04494873	chr5	72677287	chr5:72676120-72678421	Island	RP11-79P5.8	TSS1500	3.1057	1.0607	4.9417	2.24E-05	0.0234	2.6034
cg11472521	chr17	48050512	chr17:48048953-48050616	Island	DLX4	1stExon	3.0505	2.3724	4.9423	2.24E-05	0.0234	2.6050
cg14476745	chr9	124979356	chr9:124979329-124979599	Island	LHX6	ExonBnd	3.9938	0.6606	4.9367	2.28E-05	0.02366	2.5905
cg18144593	chr2	71116411	chr2:71115927-71116412	Island	AC007040.5	5'UTR	3.9329	1.1426	4.9271	2.34E-05	0.02385	2.5660
cg22927510	chr22	50968328	chr22:50963608-50970768	Island	TYMP;TYMP;TYMP;TYMP	5'UTR;5'UTR;5'UTR;5'UTR	2.6876	4.5647	4.9271	2.34E-05	0.02385	2.5660
cg18552861	chr2	20865845	chr2:20865289-20867589	Island	GDF7	TSS1500	3.2567	3.9270	4.9067	2.49E-05	0.0247	2.5135
cg02948624	chr22	19743759	chr22:19742901-19744729	Island	TBX1	TSS1500	3.8634	2.6965	4.8980	2.55E-05	0.02512	2.4912
cg12788467	chr17	36105335	chr17:36105334-36105583	Island	RP11-115K3.1;RP11-115K3.1;HNF1B;HNF1B;HNF1B	1stExon;5'UTR;TSS1500;TSS200;TSS1500	3.3293	2.2269	4.8873	2.63E-05	0.02556	2.4639
cg02380585	chr17	33776683	chr17:33776553-33776888	Island	SLFN13;SLFN13;SLFN13;SLFN13	TSS1500;TSS1500;TSS1500;TSS1500	4.4145	1.6620	4.8850	2.65E-05	0.02568	2.4579
cg13393721	chr1	43815035	chr1:43814305-43815277	Island	MPL;MPL	3'UTR;ExonBnd	-1.7275	1.9152	-4.8842	2.65E-05	0.02568	2.4558
cg02157015	chr9	100615244	chr9:100615234-100617510	Island	FOXE1	TSS1500	2.8972	3.6878	4.8777	2.71E-05	0.02597	2.4391
cg02049734	chr2	31806284	chr2:31805293-31806403	Island	SRD5A2	TSS200	1.7403	0.2954	4.8685	2.78E-05	0.02609	2.4157
cg16236507	chr17	48050079	chr17:48048953-48050616	Island	DLX4	TSS200	4.2249	3.7443	4.8677	2.78E-05	0.02609	2.4137
cg21910543	chr22	19744062	chr22:19742901-19744729	Island	TBX1	TSS200	4.0897	0.8058	4.8691	2.77E-05	0.02609	2.4171
cg13306522	chr5	54519594	chr5:54519054-54519628	Island	RP11-528L24.3	3'UTR	2.3214	0.8329	4.8586	2.86E-05	0.02646	2.3903
cg17424452	chr2	45165499	chr2:45164561-45166567	Island	RP11-89K21.1;RP11-89K21.1	3'UTR;3'UTR	3.2504	3.0489	4.8541	2.90E-05	0.02663	2.3787
cg13604794	chr17	48050338	chr17:48048953-48050616	Island	DLX4;DLX4	5'UTR;1stExon	2.3131	2.7402	4.8433	2.99E-05	0.02707	2.3509
cg22876812	chr2	71116188	chr2:71115927-71116412	Island	AC007040.5	5'UTR	5.5430	2.5686	4.8415	3.01E-05	0.02715	2.3465
cg17092594	chr9	100615349	chr9:100615234-100617510	Island	FOXE1	TSS200	3.8549	4.6659	4.8358	3.06E-05	0.02747	2.3318
cg13808083	chr9	96716527	chr9:96713326-96718186	Island	BARX1;RP11-231K24.2	TSS1500;TSS1500	3.3044	1.3169	4.8340	3.07E-05	0.02748	2.3272
cg25973534	chr5	5139853	chr5:5139441-5140941	Island	CTD-2297D10.2;ADAMTS16	3'UTR;TSS1500	3.5649	3.7381	4.8335	3.08E-05	0.02748	2.3258
cg13216999	chr2	74730166	chr2:74729399-74731166	Island	RP11-523H20.2;RP11-523H20.2;LBX2;LBX2;LBX2;LBX2;LBX2	1stExon;5'UTR;1stExon;1stExon;5'UTR;TSS200;3'UTR	1.9192	3.1066	4.8290	3.12E-05	0.02767	2.3144
cg09412931	chr8	17104627	chr8:17103890-17104863	Island	VPS37A;VPS37A;VPS37A;VPS37A;VPS37A;VPS37A;CNOT7;CNOT7	5'UTR;1stExon;5'UTR;1stExon;TSS200;TSS200;TSS1500;TSS1500	1.6625	4.2819	4.8212	3.19E-05	0.02819	2.2943
cg10253457	chr15	101460077	chr15:101458286-101460223	Island	RP11-66B24.4;LRRK1;LRRK1;LRRK1;RP11-66B24.4;RP11-66B24.4;LRRK1	TSS1500;5'UTR;5'UTR;5'UTR;TSS1500;TSS1500;5'UTR	2.2675	3.9010	4.8194	3.21E-05	0.02827	2.2899
cg09411999	chr7	27214383	chr7:27212416-27214396	Island	HOXA10;HOXA10;RP1-170O19.21;RP1-170O19.21	TSS1500;TSS1500;1stExon;3'UTR	3.2418	1.1273	4.8185	3.22E-05	0.02833	2.2873
cg19686940	chr10	128193332	chr10:128193311-128193531	Island	C10orf90	3'UTR	-1.8996	-2.1776	-4.8169	3.23E-05	0.0284	2.2832
cg07050616	chr10	102996197	chr10:102996034-102996646	Island	RP11-107I14.1;RP11-107I14.1	1stExon;5'UTR	3.2618	2.9334	4.8162	3.24E-05	0.02842	2.2816
cg07143083	chr7	70597921	chr7:70596228-70598382	Island	WBSCR17	1stExon	3.4459	3.7641	4.8118	3.28E-05	0.02865	2.2703
cg17440907	chr2	101437415	chr2:101434979-101437453	Island	NPAS2;NPAS2	TSS200;5'UTR	2.2395	3.8832	4.8030	3.37E-05	0.02903	2.2478
cg09723488	chr9	124987896	chr9:124987743-124991086	Island	LHX6	3'UTR	1.7793	0.5270	4.7958	3.44E-05	0.02924	2.2294
cg15444947	chr7	28996491	chr7:28995305-28998541	Island	TRIL;AC005013.5;AC005013.5	1stExon;TSS1500;5'UTR	-4.2721	0.6804	-4.7955	3.44E-05	0.02924	2.2284
cg19957905	chr2	119592605	chr2:119592602-119593845	Island	RP11-19E11.1;RP11-19E11.1	5'UTR;1stExon	4.2575	2.4715	4.7829	3.57E-05	0.03004	2.1961
cg23005885	chr15	90543450	chr15:90543410-90543835	Island	ZNF710	TSS1500	-3.1897	1.9139	-4.7802	3.60E-05	0.03018	2.1894
cg03087610	chr10	71892362	chr10:71892092-71892802	Island	AIFM2;AIFM2	5'UTR;5'UTR	-3.6903	0.9974	-4.7773	3.63E-05	0.03026	2.1819
cg08039704	chr17	48047123	chr17:48045788-48047207	Island	DLX4	5'UTR	2.8085	4.3796	4.7782	3.62E-05	0.03026	2.1842
cg20436037	chr20	33879989	chr20:33879904-33880215	Island	FAM83C	1stExon	-2.0450	-3.6098	-4.7777	3.62E-05	0.03026	2.1828
cg16068669	chr7	143599340	chr7:143598867-143599426	Island	FAM115A;FAM115A	TSS200;TSS200	1.6987	3.5494	4.7751	3.65E-05	0.03028	2.1763
cg21237418	chr17	27045043	chr17:27044168-27045049	Island	RAB34;RAB34;RAB34;RPL23A;RAB34;RAB34	5'UTR;5'UTR;TSS200;TSS1500;TSS200;TSS1500	-2.9992	-0.1080	-4.7738	3.67E-05	0.03031	2.1728
cg07201017	chr10	102996571	chr10:102996034-102996646	Island	RP11-107I14.1;RP11-107I14.1	1stExon;5'UTR	3.2065	1.5112	4.7729	3.68E-05	0.03033	2.1706
cg05899618	chr2	20865847	chr2:20865289-20867589	Island	GDF7	TSS1500	2.9786	3.9877	4.7722	3.68E-05	0.03036	2.1689
cg19378036	chr17	36105364	chr17:36105334-36105583	Island	RP11-115K3.1;RP11-115K3.1;HNF1B;HNF1B;HNF1B	1stExon;5'UTR;TSS1500;TSS200;TSS1500	3.7992	2.0725	4.7704	3.70E-05	0.03045	2.1643
cg08793877	chr2	71115994	chr2:71115927-71116412	Island	AC007040.5	5'UTR	3.7085	2.3855	4.7636	3.78E-05	0.03071	2.1469
cg08825084	chr2	119602862	chr2:119602616-119604486	Island	EN1	TSS1500	1.9170	2.4004	4.7613	3.80E-05	0.03075	2.1408
cg22796507	chr1	47882739	chr1:47881896-47883065	Island	FOXE3	1stExon	3.7984	4.0969	4.7616	3.80E-05	0.03075	2.1418
cg06080793	chr6	1625457	chr6:1624185-1625468	Island	GMDS;GMDS	3'UTR;1stExon	-3.3262	0.8160	-4.7417	4.03E-05	0.03196	2.0908
cg26472572	chr17	27045022	chr17:27044168-27045049	Island	RAB34;RAB34;RAB34;RPL23A;RAB34;RAB34	5'UTR;5'UTR;TSS200;TSS1500;TSS200;TSS1500	-3.2411	0.3333	-4.7416	4.03E-05	0.03196	2.0904
cg09672187	chr5	1885367	chr5:1881924-1887743	Island	IRX4;IRX4	5'UTR;5'UTR	3.5225	0.5232	4.7401	4.04E-05	0.03197	2.0867
cg13974394	chr5	1882188	chr5:1881924-1887743	Island	IRX4;IRX4	TSS1500;ExonBnd	2.8525	2.7629	4.7370	4.08E-05	0.0321	2.0788
cg22697962	chr2	71192118	chr2:71192094-71192495	Island	AC007040.11	3'UTR	2.3299	2.7504	4.7321	4.14E-05	0.03233	2.0661
cg25806347	chr2	45164720	chr2:45164561-45166567	Island	RP11-89K21.1;RP11-89K21.1	3'UTR;3'UTR	3.2527	3.0401	4.7315	4.15E-05	0.03233	2.0647
cg11815914	chr18	59992350	chr18:59992069-59993556	Island	TNFRSF11A;TNFRSF11A	TSS200;TSS200	3.2343	2.9575	4.7195	4.29E-05	0.03307	2.0340
cg08555653	chr3	138659021	chr3:138656627-138659107	Island	RP11-548O1.3	3'UTR	2.8428	1.8263	4.7172	4.32E-05	0.0332	2.0280
cg22727572	chr2	63272334	chr2:63272127-63272409	Island	AC009501.4;EHBP1;AC009501.4;EHBP1;AC009501.4	3'UTR;ExonBnd;3'UTR;5'UTR;3'UTR	-2.7729	-3.0929	-4.7069	4.46E-05	0.03368	2.0018
cg01274742	chr17	27045030	chr17:27044168-27045049	Island	RAB34;RAB34;RAB34;RPL23A;RAB34;RAB34	5'UTR;5'UTR;TSS200;TSS1500;TSS200;TSS1500	-2.7365	-0.1167	-4.7033	4.50E-05	0.03387	1.9926

cg15393275	chr2	45165193	chr2:45164561-45166567	Island	RP11-89K21.1;RP11-89K21.1	3'UTR;3'UTR	3.9161	2.2649	4.6990	4.56E-05	0.03406	1.9815
cg01485075	chr19	22817371	chr19:22817274-22817546	Island	ZNF492	5'UTR	2.1566	3.8125	4.6986	4.57E-05	0.03407	1.9804
cg13518883	chr2	239755896	chr2:239755096-239758310	Island	TWIST2	TSS1500	3.7640	2.8642	4.6848	4.75E-05	0.03483	1.9453
cg11345438	chr1	11986397	chr1:11985643-11986634	Island	KIAA2013;KIAA2013;KIAA2013;KIAA2013	1stExon;1stExon;5'UTR;5'UTR	-1.0186	0.0011	-4.6835	4.77E-05	0.03493	1.9420
cg26095658	chr2	71017887	chr2:71017028-71017976	Island	FIGLA	TSS200	3.2203	1.3506	4.6802	4.82E-05	0.03506	1.9334
cg15368905	chr6	78172337	chr6:78172231-78174088	Island	HTR1B	1stExon	3.6398	4.0529	4.6759	4.88E-05	0.03532	1.9224
cg06831953	chr6	1391249	chr6:1389139-1391393	Island	FOXF2	1stExon	1.4833	4.1975	4.6754	4.88E-05	0.03532	1.9213
cg14091208	chr3	128722579	chr3:128722283-128723036	Island	KIAA1257;CCDC48	TSS1500;5'UTR	2.7489	1.4914	4.6624	5.07E-05	0.03617	1.8881
cg06526872	chr1	44399363	chr1:44399068-44399535	Island	ARTN;ARTN;ARTN;ARTN;ARTN	TSS1500;TSS1500;1stExon;TSS200;5'UTR	-1.9109	-0.3286	-4.6517	5.23E-05	0.03659	1.8606
cg03309232	chr16	216626	chr16:214343-216720	Island	HBM	5'UTR	3.1297	4.1425	4.6489	5.28E-05	0.03668	1.8536
cg20599967	chr2	119592754	chr2:119592602-119593845	Island	RP11-19E11.1	TSS200	3.2373	1.4942	4.6356	5.48E-05	0.0373	1.8194
cg13641903	chr11	32452608	chr11:32452144-32452708	Island	WT1	TSS1500	3.3349	1.7445	4.6322	5.54E-05	0.03744	1.8110
cg27552679	chr15	74425757	chr15:74425091-74428821	Island	ISLR2	5'UTR	4.6607	1.2800	4.6230	5.69E-05	0.03822	1.7873
cg16722536	chr22	19744067	chr22:19742901-19744729	Island	TBX1	TSS200	2.6248	1.3794	4.6213	5.72E-05	0.03824	1.7830
cg23898837	chr2	119592756	chr2:119592602-119593845	Island	RP11-19E11.1	TSS200	4.0184	2.2720	4.6203	5.73E-05	0.03824	1.7806
cg00964321	chr16	15083956	chr16:15083366-15084045	Island	PDXDC1;PDXDC1	5'UTR;5'UTR	-1.2573	1.0536	-4.6187	5.76E-05	0.03839	1.7764
cg07685512	chr15	74425884	chr15:74425091-74428821	Island	ISLR2	5'UTR	3.5465	1.1121	4.6129	5.86E-05	0.03881	1.7615
cg25090972	chr7	156797988	chr7:156795355-156799394	Island	RP5-1121A15.4;MNX1;MNX1;MNX1	TSS1500;3'UTR;3'UTR;3'UTR	2.5103	3.2313	4.6097	5.91E-05	0.03905	1.7535
cg08036502	chr15	74420511	chr15:74419870-74423044	Island	RP11-247C2.2;RP11-247C2.2;RP11-247C2.2;ISLR2;ISLR2;ISLR2	3'UTR;1stExon;3'UTR;TSS1500;TSS1500;TSS1500;5'UTR	3.8220	2.8377	4.6071	5.96E-05	0.03917	1.7469
cg00700487	chr7	150706128	chr7:150705759-150706129	Island	NOS3	TSS1500	-1.6346	-1.0476	-4.6038	6.02E-05	0.0392	1.7385
cg01663745	chr11	94706857	chr11:94706291-94707060	Island	CWC15;KDM4D;KDM4D;KDM4D	TSS200;TSS200;1stExon;5'UTR	-3.0145	5.8485	-4.6036	6.02E-05	0.0392	1.7378
cg09713234	chr10	28035157	chr10:28030182-28035211	Island	MKX;MKX;RP11-360I20.2	TSS1500;TSS200;5'UTR	2.0220	2.5911	4.6003	6.08E-05	0.03933	1.7295
cg24153044	chr1	6485201	chr1:6484503-6485327	Island	ESPN;HES2	1stExon;TSS1500	3.3193	2.0166	4.5996	6.09E-05	0.03939	1.7276
cg15382538	chr5	72678193	chr5:72676120-72678421	Island	RP11-79P5.8	TSS200	3.7463	3.2422	4.5955	6.16E-05	0.03959	1.7172
cg11687330	chr11	132953239	chr11:132952538-132953307	Island	OPCML	3'UTR	-3.8037	-0.0535	-4.5805	6.44E-05	0.04023	1.6791
cg17441018	chr17	48050225	chr17:48048953-48050616	Island	DLX4;DLX4	5'UTR;1stExon	2.8766	1.6472	4.5796	6.45E-05	0.04027	1.6767
cg00142072	chr11	32452455	chr11:32452144-32452708	Island	WT1	TSS200	2.9948	1.1324	4.5786	6.47E-05	0.04034	1.6742
cg00773413	chr10	77168431	chr10:77155128-77169600	Island	ZNF503-AS2	3'UTR	3.8442	3.3006	4.5758	6.53E-05	0.04051	1.6671
cg26144458	chr21	38070148	chr21:38068193-38073891	Island	SIM2;SIM2	TSS1500;TSS1500	2.3794	1.5521	4.5654	6.73E-05	0.04127	1.6406
cg03313364	chr17	40440904	chr17:40440188-40441014	Island	STAT5A;STAT5A;STAT5A;STAT5A	5'UTR;5'UTR;5'UTR;5'UTR	0.9186	-0.5753	4.5642	6.75E-05	0.04136	1.6374
cg24826696	chr10	129534722	chr10:129534410-129537366	Island	FOXI2	TSS1500	2.9958	2.8341	4.5588	6.86E-05	0.04163	1.6235
cg11238564	chr16	67218787	chr16:67217716-67219219	Island	KIAA0895L;KIAA0895L;KIAA0895L;EXOC3L1	TSS1500;TSS1500;TSS1500;ExonBnd	-1.7851	0.0524	-4.5536	6.96E-05	0.0419	1.6104
cg20547606	chr20	30639933	chr20:30639908-30640786	Island	HCK;HCK;HCK;HCK	TSS200;TSS200;TSS200;TSS200	2.4610	3.8019	4.5455	7.13E-05	0.04238	1.5898
cg02100629	chr10	71892760	chr10:71892092-71892802	Island	AIFM2;AIFM2	TSS200;TSS200	-2.3975	2.0781	-4.5434	7.17E-05	0.04239	1.5843
cg12500879	chr11	31848649	chr11:31848487-31848776	Island	RCN1	5'UTR	-5.2964	-0.1975	-4.5409	7.22E-05	0.0425	1.5782
cg02172150	chr7	70597774	chr7:70596228-70598382	Island	WBSCR17;WBSCR17	5'UTR;1stExon	2.5638	4.4068	4.5385	7.27E-05	0.04254	1.5720
cg04105250	chr2	171679114	chr2:171678546-171680358	Island	GAD1	5'UTR	3.0031	0.9511	4.5384	7.28E-05	0.04254	1.5716
cg12828331	chr3	42543469	chr3:42542820-42544524	Island	VIPR1;VIPR1;VIPR1;VIPR1	TSS1500;TSS1500;TSS1500;5'UTR	2.8189	3.9425	4.5326	7.40E-05	0.04267	1.5570
cg24853724	chr7	28997403	chr7:28995305-28998541	Island	TRIL;AC005013.5;AC005013.5;AC005013.5	1stExon;5'UTR;1stExon;5'UTR	-3.6003	1.7561	-4.5335	7.38E-05	0.04267	1.5592
cg15750546	chr21	38080975	chr21:38079941-38081833	Island	SIM2	5'UTR	2.5853	2.6330	4.5304	7.45E-05	0.04283	1.5513
cg20860638	chr3	196756715	chr3:196755677-196757374	Island	MFI2;MFI2	TSS200;TSS200	2.1396	4.6255	4.5256	7.55E-05	0.04308	1.5390
cg07952270	chr10	102996510	chr10:102996034-102996646	Island	RP11-107I14.1;RP11-107I14.1	1stExon;5'UTR	2.4341	0.7588	4.5220	7.63E-05	0.04311	1.5299
cg23325963	chr2	45160093	chr2:45159893-45160112	Island	RP11-89K21.1;RP11-89K21.1;RP11-89K21.1;RP11-89K21.1	3'UTR;3'UTR;3'UTR;3'UTR	4.6978	3.0799	4.5204	7.66E-05	0.04315	1.5260
cg22125805	chr12	119212248	chr12:119212110-119212393	Island	RP11-357K6.1	3'UTR	1.9568	5.3939	4.5200	7.67E-05	0.04318	1.5248
cg01281911	chr1	47882686	chr1:47881896-47883065	Island	FOXE3	1stExon	3.2041	3.7388	4.5171	7.74E-05	0.04339	1.5175
cg15989981	chr13	20736009	chr13:20735043-20736157	Island	GJA3	TSS1500	3.4055	1.3162	4.5156	7.77E-05	0.04355	1.5137
cg09437522	chr20	57431202	chr20:57429024-57431239	Island	GNAS;GNAS;GNAS;GNAS	3'UTR;3'UTR;3'UTR;5'UTR	1.5066	-0.1290	4.5128	7.83E-05	0.04379	1.5066
cg06161964	chr5	1884513	chr5:1881924-1887743	Island	IRX4;IRX4;CTD-2194D22.3	5'UTR;5'UTR;5'UTR	3.9326	2.3183	4.5093	7.91E-05	0.04392	1.4977
cg16508480	chr5	5139874	chr5:5139441-5140941	Island	CTD-2297D10.2;ADAMTS16	3'UTR;TSS1500	3.2388	3.1176	4.5071	7.97E-05	0.04406	1.4919
cg15885672	chr17	59488116	chr17:59488115-59490485	Island	C17orf82	TSS1500	3.0098	1.4716	4.5056	8.00E-05	0.04414	1.4882
cg05387167	chr17	46629804	chr17:46629553-46629816	Island	HOXB3;HOXB3;HOXB3;HOXB-AS3;HOXB3;HOXB3	1stExon;5'UTR;5'UTR;5'UTR;5'UTR;5'UTR	-3.1395	0.1381	-4.4905	8.36E-05	0.04471	1.4498
cg09460082	chr1	149287435	chr1:149286812-149287773	Island	U1;RP11-403I13.8	TSS1500;TSS200	1.1201	0.1073	4.4904	8.36E-05	0.04471	1.4494
cg02227453	chr22	50919482	chr22:50919453-50920376	Island	ADM2;ADM2;ADM2	TSS1500;TSS1500;TSS1500	3.1135	1.4637	4.4886	8.40E-05	0.04479	1.4451
cg13242000	chr15	74426086	chr15:74425091-74428821	Island	ISLR2	5'UTR	3.0925	1.1177	4.4889	8.40E-05	0.04479	1.4458
cg14594607	chr8	10588833	chr8:10587304-10591109	Island	SOX7;CTD-2135J3.3	TSS1500;5'UTR	2.2930	0.9594	4.4881	8.42E-05	0.04479	1.4437
cg02624770	chr7	28997137	chr7:28995305-28998541	Island	TRIL;AC005013.5;AC005013.5	1stExon;5'UTR;TSS200	-3.4070	0.1302	-4.4868	8.45E-05	0.04481	1.4404
cg19501982	chr4	190938265	chr4:190938106-190938848	Island	RN5S175	TSS1500	3.0614	2.9361	4.4864	8.46E-05	0.04483	1.4394
cg04594191	chr7	28997156	chr7:28995305-28998541	Island	TRIL;AC005013.5;AC005013.5	1stExon;5'UTR;TSS200	-2.4933	0.3670	-4.4793	8.63E-05	0.04519	1.4213
cg15774864	chr2	63272214	chr2:63272127-63272409	Island	AC009501.4;EHBP1;EHBP1;AC009501.4	3'UTR;ExonBnd;5'UTR;3'UTR	-2.1713	-3.6007	-4.4758	8.72E-05	0.04539	1.4124
cg21513542	chrX	49042807	chrX:49042676-49043059	Island	PRICKLE3;PRICKLE3;PRICKLE3;PRICKLE3;PRICKLE3;PRICKLE3;PRICKLE3;PRICKL	TSS1500;TSS1500;1stExon;1stExon;1stExon;5'UTR;5'UTR;5'UTR	-4.6920	0.6209	-4.4670	8.95E-05	0.0459	1.3901
cg03476000	chr1	6480167	chr1:6479057-6480938	Island	HES2;HES2;HES2;HES2	5'UTR;TSS200;TSS1500;TSS1500	1.8578	2.9732	4.4610	9.10E-05	0.04607	1.3749
cg03998104	chr2	74743664	chr2:74740455-74743795	Island	TLX2	3'UTR	3.1751	2.5797	4.4599	9.13E-05	0.04607	1.3719
cg19048532	chr7	27148002	chr7:27147589-27148389	Island	HOXA-AS2	5'UTR	-2.2284	0.4978	-4.4614	9.09E-05	0.04607	1.3760
cg23634124	chr17	48049952	chr17:48048953-48050616	Island	DLX4	TSS200	3.4172	3.5028	4.4609	9.10E-05	0.04607	1.3747
cg07545278	chr16	67219152	chr16:67217716-67219219	Island	KIAA0895L;KIAA0895L;KIAA0895L;EXOC3L1	TSS1500;TSS1500;TSS1500;ExonBnd	-2.3702	-0.3318	-4.4556	9.25E-05	0.04645	1.3611
cg04095339	chr5	1879917	chr5:1879689-1879928	Island	IRX4	3'UTR	2.5004	-0.0219	4.4511	9.37E-05	0.04682	1.3497
cg24226687	chr20	60757891	chr20:60757609-60758378	Island	GTPBP5;GTPBP5;GTPBP5	TSS1500;TSS1500;TSS1500	1.6279	5.4748	4.4490	9.42E-05	0.04692	1.3444
cg05553634	chr1	53308344	chr1:53308294-53309262	Island	ZYG11A;ZYG11A;ZYG11A	1stExon;TSS200;5'UTR	2.1861	5.2354	4.4464	9.50E-05	0.04706	1.3378

cg23618344	chr12	54423428	chr12:54423427-54423712	Island	HOXC4;HOXC5	5'UTR;5'UTR	1.7558	3.6848	4.4407	9.66E-05	0.04738	1.3232
cg12338417	chr3	32860178	chr3:32858194-32860506	Island	TRIM71	1stExon	4.9678	3.8600	4.4283	0.0001	0.04813	1.2919
cg03206359	chr5	172663793	chr5:172663616-172664584	Island	NKX2-5	TSS1500	2.8349	2.1806	4.4207	0.0001	0.04857	1.2725
cg22132508	chr3	138658878	chr3:138656627-138659107	Island	RP11-548O1.3	3'UTR	2.2467	2.1449	4.4205	0.0001	0.04857	1.2721
cg13441766	chr5	134376442	chr5:134374385-134376751	Island	CTC-276P9.1;CTC-276P9.1;CTC-203F4.1;CTC-349C3.1;CTC-349C3.1	TSS1500;TSS1500;5'UTR;5'UTR;5'UTR	1.2663	4.4885	4.4202	0.0001	0.04858	1.2713
cg07211474	chr5	114515104	chr5:114514716-114516220	Island	TRIM36	5'UTR	1.5131	0.4845	4.4198	0.0001	0.04859	1.2703

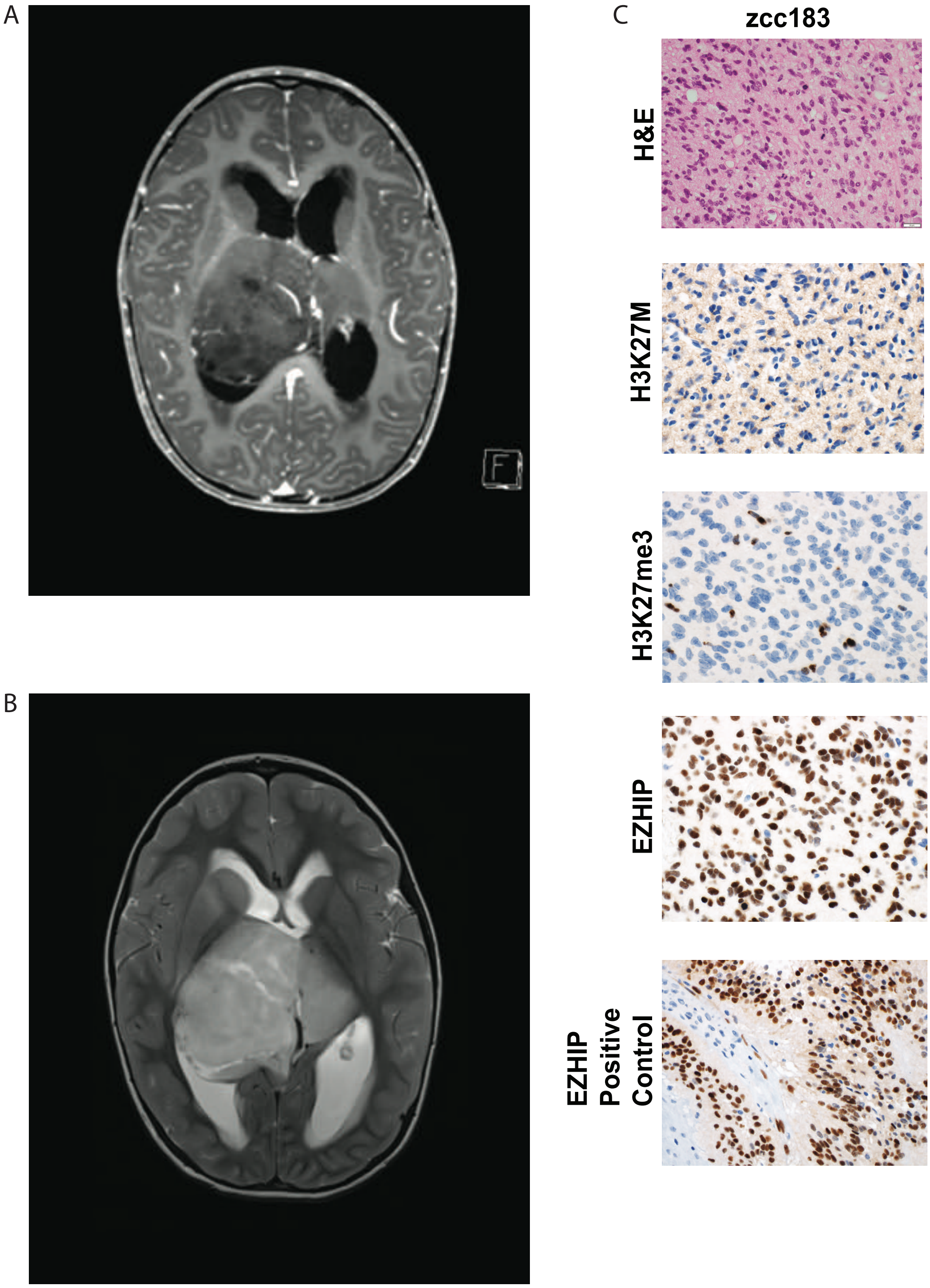
A
B
C
zcc183
H&E
H3K27M
H3K27me3
EZHIP
EZHIP
Positive
Control

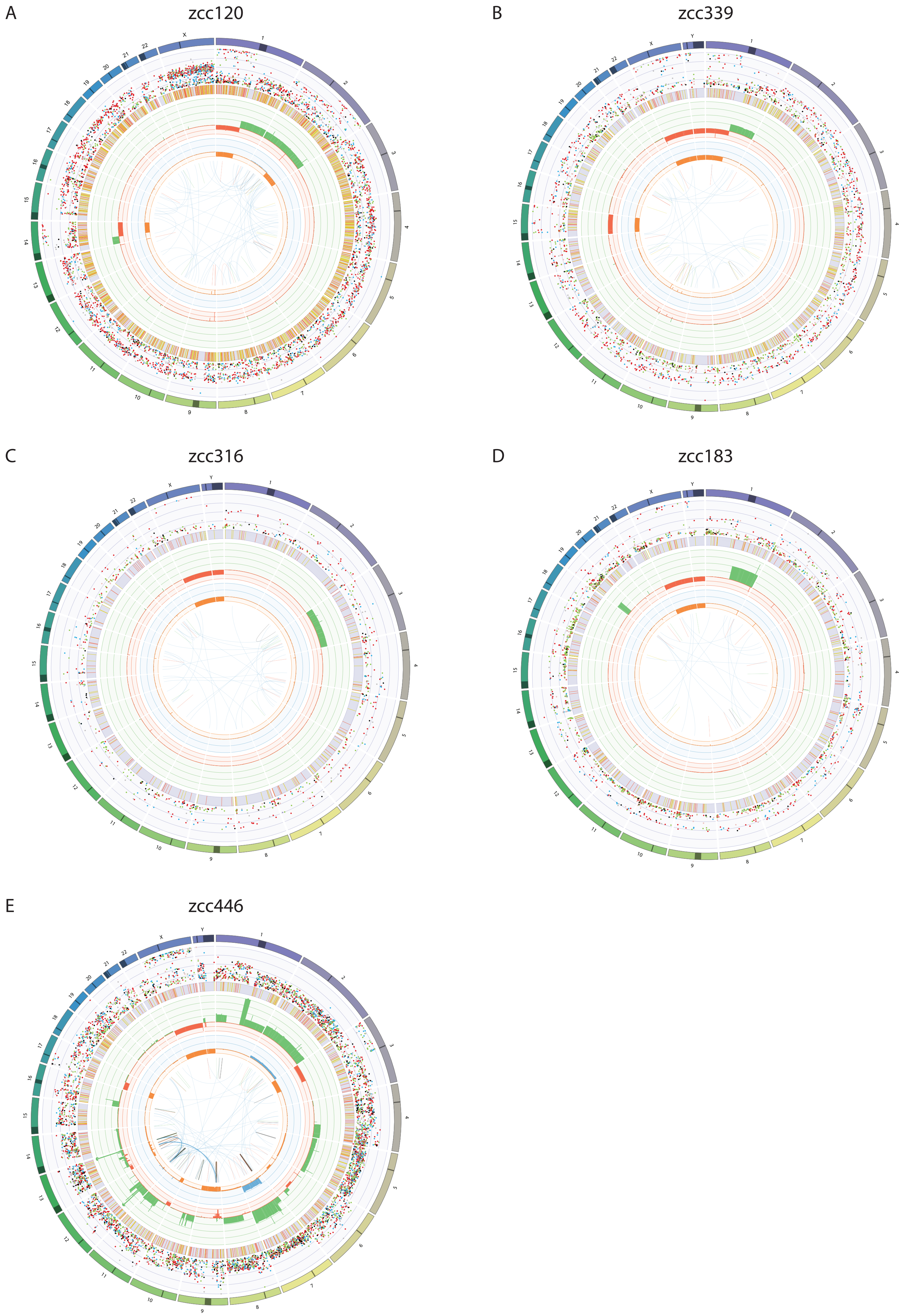
A
zcc120
B
zcc339
C
zcc316
D
zcc183
E
zcc446

A

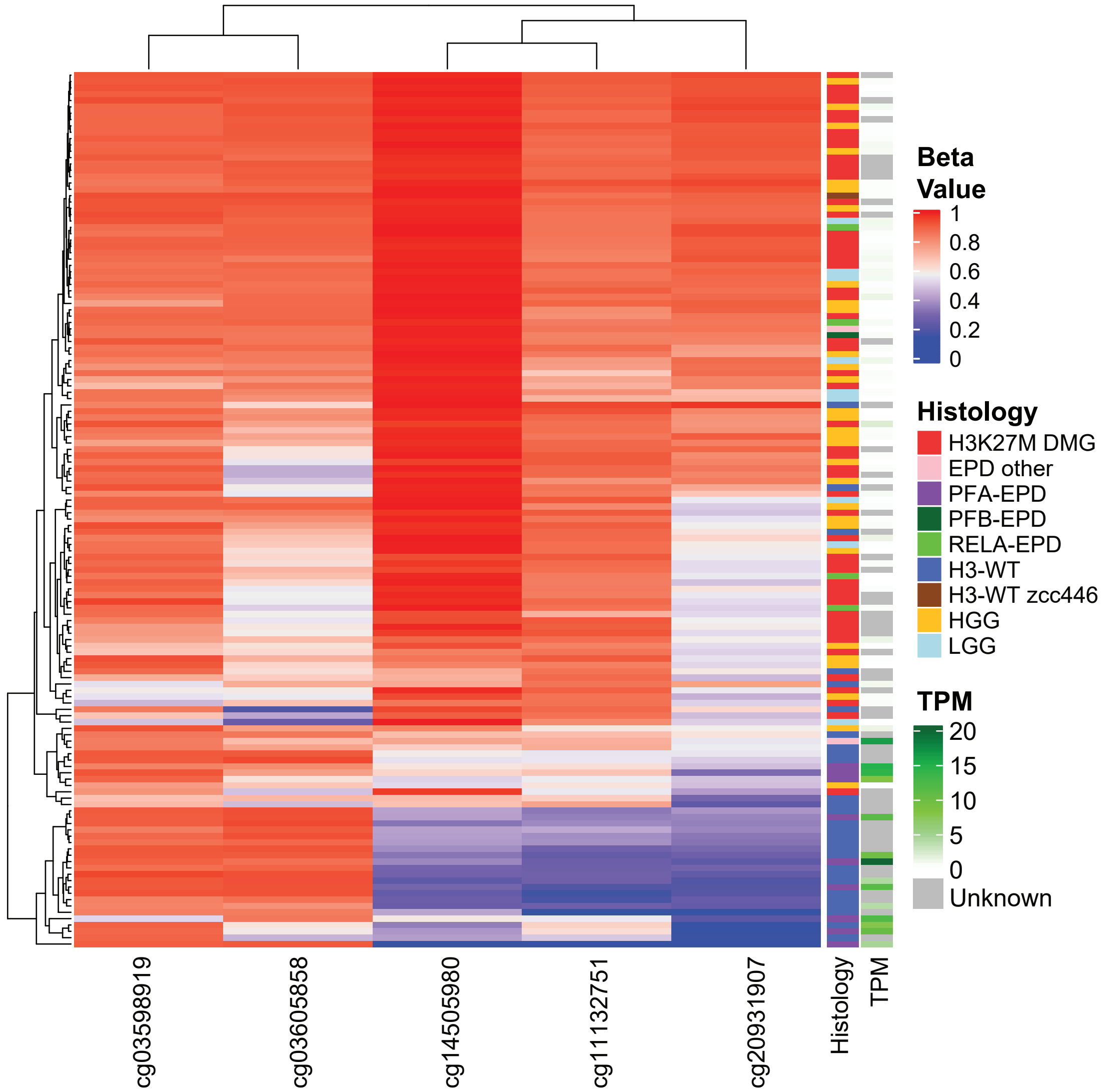
Beta Value
1
0.8
0.6
0.4
0.2
0
Histology
H3K27M DMG
EPD other
PFA-EPD
PFB-EPD
RELA-EPD
H3-WT
H3-WT zcc446
HGG
LGG
TPM
20
15
10
5
0
Unknown
cg03598919
cg03605858
cg14505980
cg11132751
cg20931907
Histology
TPM

B

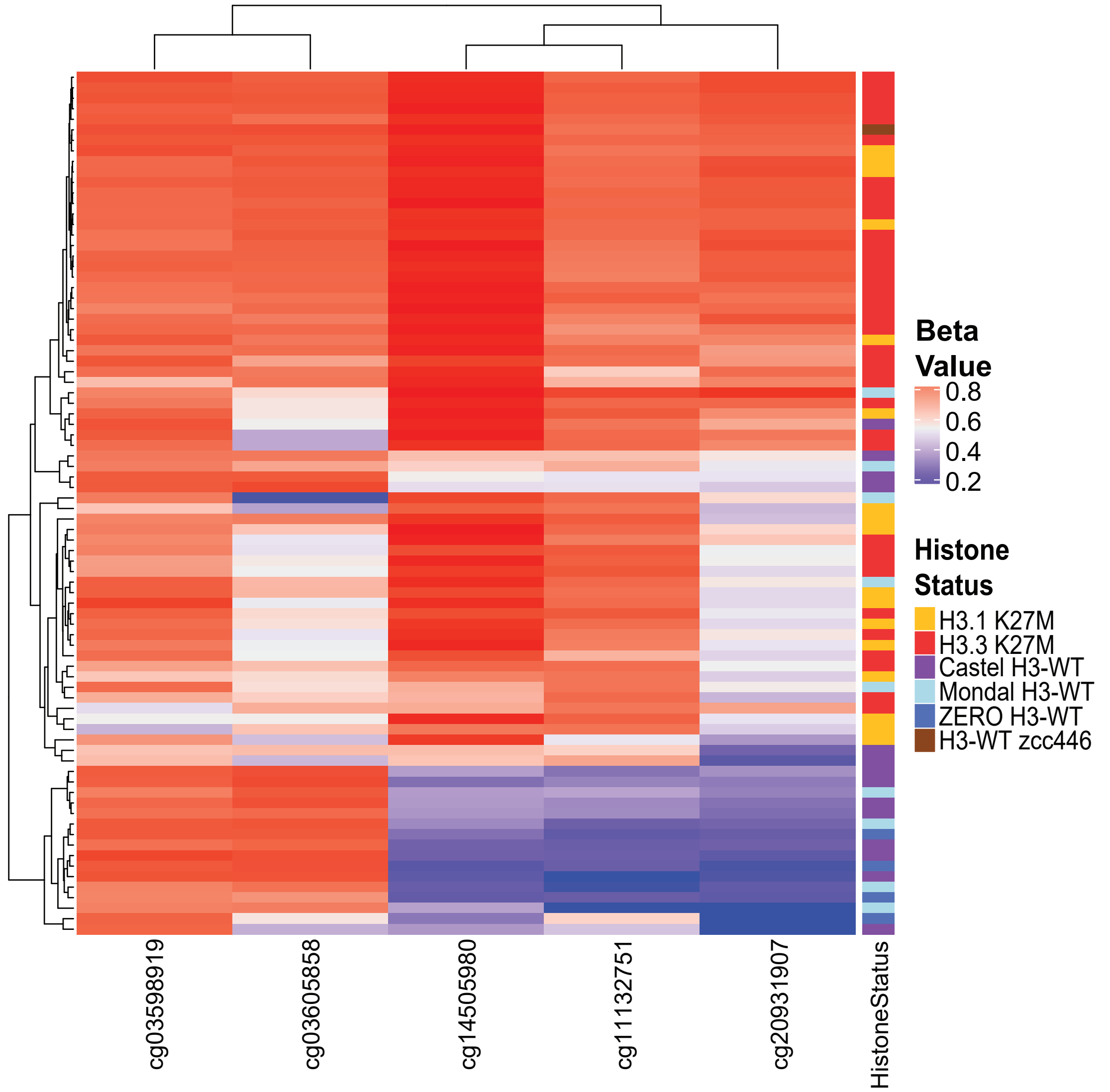
Beta Value
0.8
0.6
0.4
0.2
Histone Status
H3.1 K27M
H3.3 K27M
Castel H3-WT
Mondal H3-WT
ZERO H3-WT
H3-WT zcc446
cg03598919
cg03605858
cg14505980
cg11132751
cg20931907
HistoneStatus
